# Argyrodite-Li_6_PS_5_Cl/Polymer-based Highly Conductive Composite Electrolyte for All-Solid-State Batteries

**DOI:** 10.1021/acsaem.3c02858

**Published:** 2024-02-16

**Authors:** Faiz Ahmed, Anna Chen, M. Virginia P. Altoé, Gao Liu

**Affiliations:** † Energy Storage and Distributed Resources Division, Lawrence Berkeley National Laboratory, Berkeley, California 94720, United States; ‡ Molecular Foundry Division, Lawrence Berkeley National Laboratory, Berkeley, California 94720, United States; § Campolindo High School, 300 Moraga Rd, Moraga, California 94556, United States

**Keywords:** solid-state-battery, ball-milling, composite electrolyte, ionic conductivity, Coulombic efficiency

## Abstract

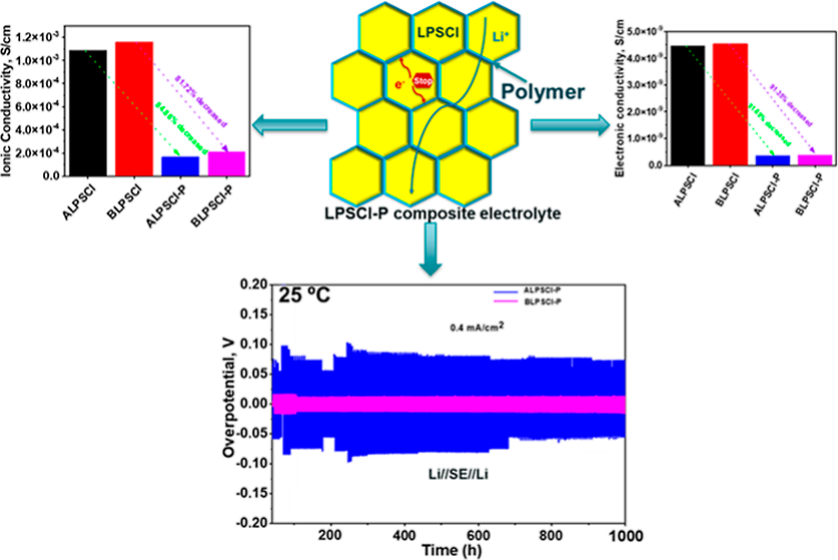

Solid-state batteries (SSBs) that incorporate the argyrodite-Li_6_PS_5_Cl (LPSCl) electrolyte hold potential as substitutes for conventional lithium-ion batteries (LIBs). However, the mismatched interface between the LPSCl electrolyte and electrodes leads to increased interfacial resistance and the rapid growth of lithium (Li) dendrites. These factors significantly impede the feasibility of their widespread industrial application. In this study, we developed a composite electrolyte of the LPSCl/polymer to enhance the contact between the electrolyte and electrodes and suppress dendrite formation at the grain boundary of the LPSCl ceramic. The monomer, triethylene glycol dimethacrylate (TEGDMA), is utilized for in situ polymerization through thermal curing to create the argyrodite LPSCl/polymer composite electrolyte. Additionally, the ball-milling technique was employed to modify the morphology and particle size of the LPSCl ceramic. The ball-milled LPSCl/polymer composite electrolyte demonstrates slightly higher ionic conductivity (ca. 2.21 × 10^–4^ S/cm) compared to the as-received LPSCl/polymer composite electrolyte (ca. 1.65 × 10^–4^ S/cm) at 25 °C. Furthermore, both composite electrolytes exhibit excellent compatibility with Li-metal and display cycling stability for up to 1000 h (375 cycles), whereas the as-received LPSCl and ball-milled LPSCl electrolytes maintain stability for up to 600 h (225 cycles) at a current density of 0.4 mA/cm^2^. The SSB with the ball-milled LPSCl/polymer composite electrolyte delivers high specific discharge capacity (138 mA h/g), Coulombic efficiency (99.97%), and better capacity retention at 0.1C, utilizing the battery configuration of coated NMC811//electrolyte//Li-Indium (In) at 25 °C.

## 1 Introduction

The intense focus of research is on all-solid-state lithium-ion batteries (ASSLBs) with solid electrolytes (SEs) owing to their potential qualities such as high energy, high power density, and enhanced safety compared to conventional LIBs.^1−3^ In spite of the numerous advantages of ASSLBs, a lot of issues still need to be solved before commercialization.^4^ Typically, SEs demonstrate lower ionic conductivity (σ) compared to liquid electrolytes.^2^ For instance, lithium phosphorus oxynitride (LiPON), a frequently employed SE, exhibits an σ of ca. 10^–6^ S/cm. In contrast, a liquid electrolyte like lithium hexafluorophosphate in ethylene carbonate and propylene carbonate demonstrates a higher σ of ca. 10^–2^ S/cm at room temperature.^2^

So far, extensive research has been conducted on SEs to address their limitations. Various approaches have been explored, including organic polymer electrolytes, inorganic ceramic electrolytes, and inorganic–organic composite electrolytes.^5−8^ However, many polymer electrolytes face challenges with modest room temperature σ due to their high degree of crystallinity and low Li-ion transference number. Such as poly(ethylene glycol) diacrylate (PEGDA), poly(ethylene oxide) (PEO), polydopamine (PDA), polyacrylonitrile (PAN), and poly(vinylidene fluoride) (PVDF) polymers exhibit low-temperature σ, worse antioxidation, and poor mechanical character.^9−15^ Furthermore, oxide-based inorganic SEs, such as LiPON, garnets, Li_1.3_Al_0.3_Ti_1.7_(PO_4_)_3_ (LATP), Li_7_La_3_Zr_2_O_12_ (LLZO), and La_0.57_Li_0.29_TiO_3_ (LLTO), have shown improved conductivity (ranging from 10^–3^ to 10^–4^ S/cm).^2,16^ Nonetheless, they are sensitive to moisture, prone to reduction during charge–discharge (CD) cycles, react with Li-metal, and tend to be brittle.^2^ However, researchers have turned to sulfide-based SEs as an alternative to oxides. Sulfide-based SEs exhibit extraordinary room temperature σ (ca. 2.5 × 10^–2^ S/cm), which equals or surpasses that of most liquid electrolytes.^17,18^ Investigations into sulfide-based SE systems, including glasses, glass ceramics, and crystalline conductors, have opened up an exciting research field on SEs. However, certain sulfide-based SEs such as LiGeP_2_S_12_ and LiSnP_2_S_12_ exhibit a strong reactivity with Li-metal, leading to the formation of dendrites at the interfaces and results in the decomposition of the SEs.^19−21^ To address this issue and mitigate side reactions between the electrolyte and Li-metal, a solution involving using Li-alloy and buffer layers between the sulfide-based SEs and Li-metal has been studied.^22−24^ Unfortunately, this approach comes with a significant drawback, as it dramatically reduces the energy density of the cells due to the low working voltage. Alternatively, sulfide-based SEs like LiPSX (X = halides), Li_3_PS_4_, and LiP_3_S_11_ have also been explored, and they display a reactivity with Li-metal, forming an ionic-conductivity interface layer.^25,26^ This layer can serve as a solid-electrolyte interface (SEI) layer, effectively suppressing side reactions and creating a smooth pathway for Li^+^ movement.^27^ Consequently, these types of sulfide-based SEs present promising options for the development of high-energy-density SSBs.

Among the various sulfide-based SEs, argyrodite LPSCl stands out as an attractive option due to its excellent σ (ca. 1.33 × 10^–3^ S/cm at room temperature) and low cost.^28^ Consequently, extensive research has been conducted on LPSCl electrolytes, yielding promising results for their applications in Li-ion and Li–S batteries.^29−34^ However, a significant challenge in using argyrodite LPSCl in ASSLBs applications is the interface instability between LPSCl and electrodes. During CD cycling, LPSCl tends to oxidize, leading to the production of elemental sulfur, polysulfides, phosphates, and lithium chloride at the electrode interface.^35,36^ These side products ultimately hinder the cycling stability and can result in dendrite formation at the interface. Another important drawback of LPSCl is its non-negligible electronic conductivity, which allows for smooth electron transport through the LPSCl electrolyte.^37^ Consequently, Li dendrites can be directly deposited at the grain boundaries of LPSCl particles, leading to a serious self-discharge issue.^37^

Recently, there has been significant use of poly(ethylene oxide) (PEO) and its derivatives-based polymers in conjunction with LPSCl to develop composite electrolytes aiming to enhance electrochemical performance, mechanical strength, and interfacial stability.^3^ Zou et al. reported on a composite electrolyte composed of PEO, lithium bis(trifluoromethanesulfonyl)imide (LiTFSI), and LPSCl, which exhibited high σ and excellent interfacial stability against Li-metal.^38^ By preventing direct contact between LPSCl and Li metal, the prepared cell demonstrated favorable Li plating-stripping ability at a current density of 0.2 mA/cm^2^ at 60 °C.^38^ Huo et al. also improved cell performance by modifying PEO, showing that a substituted terminal group of poly(ethylene glycol) not only stabilized inner interfaces but also extended the electrochemical window of the composite electrolyte.^39^ Despite the advantages of the aforementioned PEO-based composite electrolytes, they are not yet suitable for practical applications due to their low σ at room temperature and dendrite formation at high current density.^3^ Addressing these challenges, researchers recently prepared composite electrolytes using only polymer and LPSCl electrolytes without the addition of any salts.^37,40,41^ Poly(*p*-phenylene oxide) (PPO), poly(ethylene glycol) dimethyl ether (PEGDME), and PVDF were used as the polymer matrix. It was observed that after the polymer was added to the LPSCl electrolyte, the voids and gaps among the LPSCl particles were filled. The polymer formed a protective layer on the LPSCl particles, reducing electronic conductivity through the LPSCl particle, ultimately suppressing dendrite formation in the grain boundary, and protecting LPSCl from moisture.^37^ As a result, the cycling performance and capacity retention of the cell with polymer/LPSCl composite electrolyte significantly increased.^34^ Therefore, the engineering of polymer/LPSCl composite electrolytes represents a promising strategy to develop dendrite and self--discharge-free, as well as humidity-stable, ASSLBs.

In this research endeavor, we developed polymer/LPSCl composite electrolytes for application in ASSLBs. The process involved utilizing the TEGDMA monomer and LPSCl ceramic as precursor materials, which were then subjected to in situ polymerization. The aim was to enhance the interfacial interaction between the SE and the electrodes. Additionally, we explored the impact of ball-milling on the particle size and morphology of LPSCl, investigating its effects on the performance of the material. The resulting composite electrolytes exhibited several advantageous properties. These included reduced sensitivity to air, exceptional σ, and diminished electronic conductivity. To assess Li plating-striping performance, Li–Li symmetric cells were employed. Notably, the Li–Li symmetric cell employing the composite electrolyte demonstrated stable cycling for a remarkable duration of over 1000 h (375 cycles) at a current density of 0.4 mA/cm^2^.

## 2 Experimental Section

### 2.1 Materials

The chemicals listed, including toluene, azobis(isobutyronitrile) (AIBN), and vapor-grown carbon fiber, were received from Sigma-Aldrich and were used in their original state without any further purification. TEGDMA was also received from Sigma-Aldrich, dried using molecular sieves, and kept inside the glovebox. Meanwhile, argyrodite LPSCl and lithium niobium oxide (LiNbO_3_) (1 wt %)-coated NMC811 cathode powder were procured from MSE Supplies in America. Furthermore, Li-metal foil, indium (In), and polypropylene separator were obtained from Albemarle and Celgard, respectively, and were kept in a glovebox for storage.

### 2.2 Instrumentations and Measurements

The chemical structures of the prepared compounds were confirmed using Fourier-transform infrared (FTIR) spectroscopy (Nicolet iS5, ASB1100426, Thermo Fisher Scientific, Massachusetts, USA). The crystallinity of the prepared SEs was examined by utilizing an X-ray diffractometer [XRD, Rigaku-Ultima (IV)] featuring Cu Kα radiation (1.5418 Å). The XRD experiment was executed over the 2θ range of 5–80°, employing a step size of 0.01°. For the XRD investigation, the specimens were safeguarded within a hermetically sealed XRD holder. A thermogravimetric analyzer (TGA, 1 STAR^e^ System from Mettler-Toledo) was used to analyze the thermal properties of the prepared SEs under an argon (Ar) atmosphere in the temperature range of (30–800) °C at a heating rate of 10 °C/min. Differential scanning calorimetry (DSC) was carried out, on a 1 STAR^e^ system from Mettler–Toledo, in the temperature range of −80 to 250 °C at a heating rate of 10 °C/min under an Ar atmosphere. The surface characteristics, elemental compositions, and topographies of the electrodes and SEs were studied through the scanning electron microscopy (SEM, JEOL JSM-750F), while X-ray photoelectron spectroscopy (XPS) was utilized for further analysis of the electrodes and SEs. To perform XPS analyses, an air-free sample holder with Ag-tape on the Si-substrate was placed inside an Ar-filled glovebox and the electrodes were secured using the Ag-tape. The XPS experiment was conducted using a Thermo Fisher K-Alpha Plus XPS/UPS analyzer (operating pressure of 2.0 × 10^–7^ Pa) with a monochromatic Al Kα X-ray (1.486 eV) source at The Molecular Foundry.

Meanwhile, electrochemical impedance spectroscopy (EIS) was performed on an impedance analyzer (Biologic, Claix, France), with an AC amplitude of 5 mV over a frequency range of 1 MHz to 100 mHz. Moreover, the electronic conductivity of the as-prepared SEs was determined by observing different current responses at varying applied voltages (0.1 to 0.5 V) as a function of time. In the model cell, stainless steel (SS)//electrolyte//stainless steel (SS), 200 mg of powder SEs were placed between two stainless steel rods and then pressed into a pellet under a pressure of 348 MPa, resulting in a diameter of 12 mm.

### 2.3 Preparation of Polymer/Ceramic Composite Electrolyte, Composite Cathode, and SSBs Cells

**Scheme 1:**
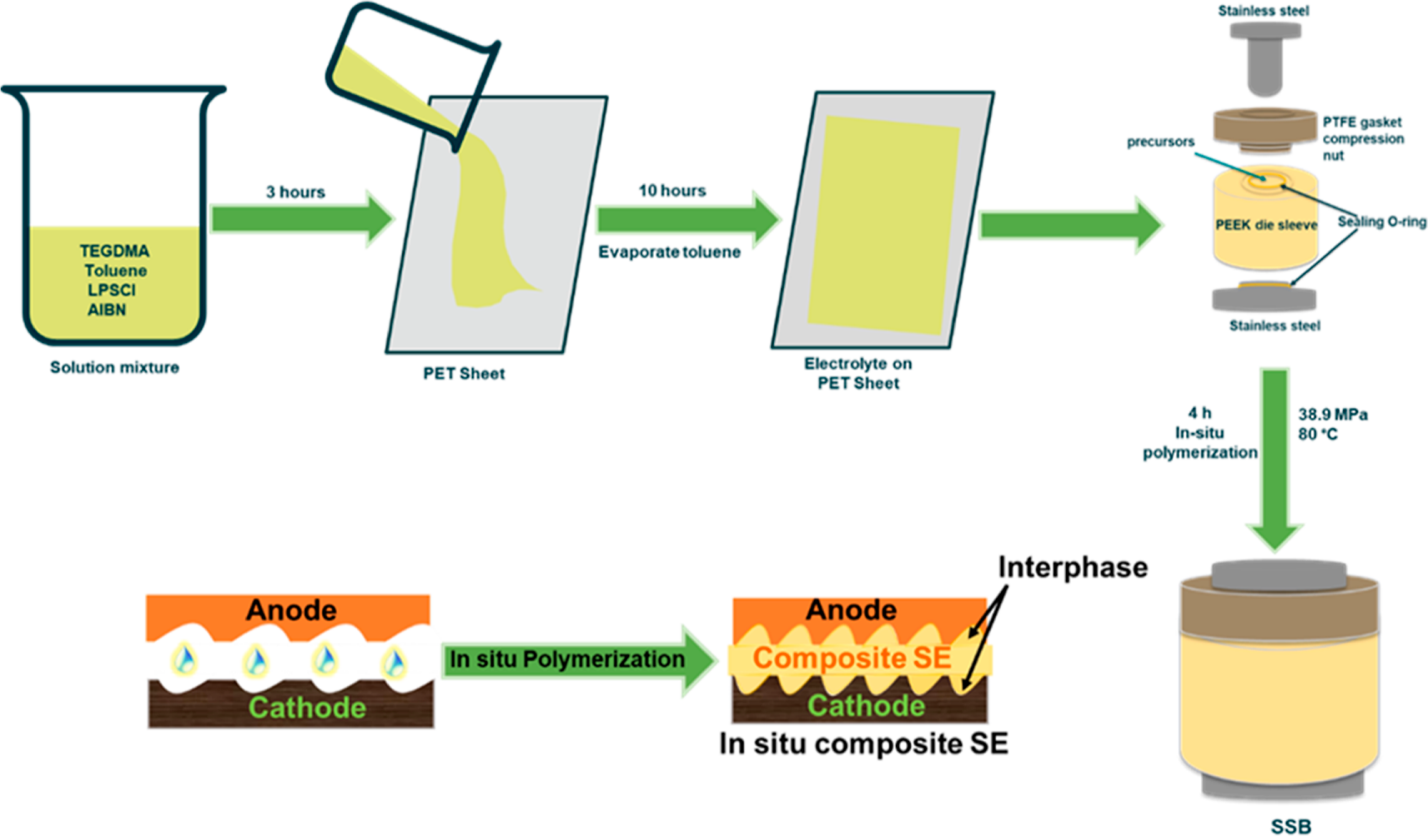
Schematic Diagram of the Preparation and In Situ Polymerization of the Polymer/Ceramic Composite Electrolyte

The fabrication and polymerization of the composite electrolytes are shown in Scheme 1. To prepare a composite electrolyte made of polymer and ceramic, TEGDMA, toluene, AIBN, and LPSCl were added. First, 0.25 g of TEGDMA and toluene (10 wt %) in a small vial were mixed and stirred for 10 min until the solution became uniform. Then, 1 g of LPSCl was added to the solution and stirred for 4 h to evenly distribute the particles. After that, AIBN (1 wt % of TEGDMA) was added to the solution and stirred for 30 min. The resulting solution was poured onto a poly(ethylene terephthalate) (PET) sheet to allow the toluene to evaporate. Once the toluene was evaporated, we placed the product into a poly(ether ether ketone) (PEEK) die sleeve and applied 348 MPa pressure. The product was then heated at 80 °C for 4 h to complete the polymerization process. The whole experiment was conducted inside an Ar-filled glovebox. In addition, to prepare ball-milled LPSCl, the as-received LPSCl was placed in a ZrO_2_ container, along with a ZrO_2_ ball, and subjected to mechanical milling using a planetary ball milling apparatus at a speed of 500 rpm for a duration of 30 min. The prepared four electrolytes; as-received LPSCl, ball-milled LPSCl, as-received LPSCl–TEGDMA polymer, and ball-milled LPSCl–TEGDMA polymer are denoted as ALPSCl, BLPSCl, ALPSCl–P, and BLPSCl–P, respectively.

For the assembly and electrochemical measurements of Li–Li symmetric cells, the electrolyte weighing 0.2 g was initially compressed at ca. 348 MPa using a PEEK die sleeve to form a pellet. Subsequently, two pieces of Li metal foil with a diameter of 10 mm were placed on either side of the electrolyte pellet and pressed with a pressure of ca. 50 MPa, and heated at 80 °C for 4 h to complete the polymerization process (for composite electrolytes). Li plating-stripping experiments were conducted at 25 °C using a Biologic (Claix, France) system under 50 MPa applied pressure. The current density were 0.4 and 0.5 mA/cm^2^. The specific operating conditions for the designated cells were mentioned in the Supporting Information section.

To prepare the cathode composite, coated NMC811 and LPSCl electrolyte were taken with the weight ratio of 80:20 in mortar inside the glovebox. Then, 2 wt % of vapor-grown carbon fiber was added and mixed properly using a pestle. For Li-coated NMC811 full cell testing, initially, 0.2 g of electrolyte was pressed into a pellet with a pressure of about 348 MPa. Next, the cathode composite material was evenly distributed on the surface of the electrolyte plate and pressed under a pressure of about 348 MPa. A Li–In (3:7, wt %) foil was then pressed onto the opposite side of the electrolyte and applied ca. 50 MPa pressure. The mass loading of the cathode composite material was approximately 12 mg. The pellet was sandwiched between two SS rods and heated at 80 °C for 4 h to complete the polymerization process. All of these processes were carried out inside an Ar-filled glovebox. The galvanostatic CD tests were conducted using a Biologic system (Claix, France) at 25 °C under 50 MPa applied pressure. The operating voltage range was from 2.4 to 4.2 V (vs Li^+^/Li).

## 3 Results and Discussion

### 3.1 Characterizations of Prepared Electrolytes

**Figure 1:**
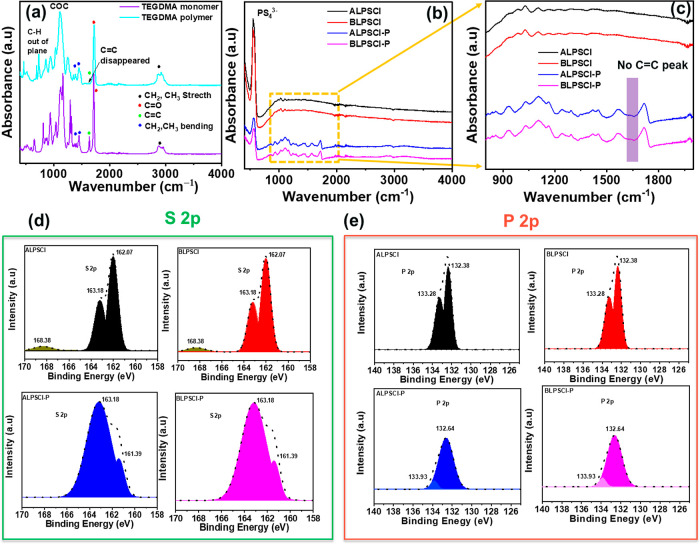
FTIR spectra of (a) TEGDMA monomer and TEGDMA polymer and (b,c) ALPSCl, BLPSCl, ALPSCl–P, and BLPSCl–P electrolytes, respectively. XPS spectra of prepared SEs: (d) S 2p and (e) P 2p.

The cross-linked polymer cannot dissolve in commonly used NMR solvents such as CDCl_3_, DMSO-*d*_6_, C_4_D_8_O, and D_2_O. Hence, FTIR spectroscopy was employed to analyze the structure of the LPSCl, TEGDMA monomer, and cross-linked TEGDMA polymer. Figure 1a–c illustrates the FTIR spectra of ALPSCl, BLPSCl, TEGDMA monomer, TEGDMA polymer, ALPSCl–P, and BLPSCl–P, respectively. In the FTIR spectrum of the TEGDMA monomer (Figure 1a), characteristic bands are observed at 3000–2850 cm^–1^ (C–H stretching), 1640 cm^–1^ (C=C stretching), 1724 cm^–1^ (C=O stretching), 1465–1375 cm^–1^ (C–H bending for −CH_2_– and −CH_3_), 1340–850 cm^–1^ (COC stretching), and 650–1000 cm^–1^ (C–H out-of-plane bend).^42^ However, upon polymerization of the TEGDMA monomer, only the C=C peaks disappear completely (Figure 1a), while the other peaks remain unchanged. This indicates the successful polymerization of TEGDMA. In Figure 1b, ALPSCl, BLPSCl, ALPSCl–P, and BLPSCl–P exhibit a characteristic FTIR band of PS_4_^–3^ at 546 cm^–1^.^43^ The C=C band is diminished in the FTIR spectrum of ALPSCl–P and BLPSCl–P (Figure 1c), indicating that LPSCl does not hinder the polymerization of the TEGDMA monomer.

The chemical states of the surface functional groups of the prepared SEs were analyzed by using XPS analysis. The corresponding deconvoluted S 2p and P 2p spectra for ALPSCl, BLPSCl, ALPSCl–P, and BLPSCl–P electrolytes are shown in Figure 1d,e, respectively. In the TEGDMA polymer XPS spectra, there were no S 2p and P 2p spectra that appeared, as shown in Figure S1. The S 2p and P 2p signals are split into two components due to spin-orbit coupling. Meanwhile, in the deconvoluted XPS spectra of the S 2p and P 2p levels in ALPSCl and BLPSCl electrolytes, clear peaks were identified at ca. (162.07 and 163.18) eV for S 2p_3/2_ and S 2p_1/2_, and at ca. (132.38 and 133.28) eV for P 2p_3/2_ and P 2p_1/2_, respectively. The observed peaks of P and S elements were associated with the PS_4_^3–^ system.^44,45^ For the ALPSCl and BLPSCl electrolytes, a second weak component at ca. 168.38 eV was detected in the S 2p spectra. This finding can be explained by traces of the sulfite environment (SO_3_^2–^) on the surface, probably due to contact with traces of oxygen.^44^ On the other hand, in the XPS spectra of ALPSCl–P and BLPSCl–P composite electrolytes, this peak was not present, suggesting that the polymer can protect the LPSCl particles from moisture and form a less air-sensitive electrolyte. Furthermore, the positions and intensities of the S 2p and P 2p peaks in ALPSCl–P and BLPSCl–P composite electrolytes exhibited slight changes, possibly attributable to the inclusion of polymer in the LPSCl ceramics.

### 3.2 Physiochemical Properties and Morphological Analyses of Prepared Electrolytes

**Figure 2:**
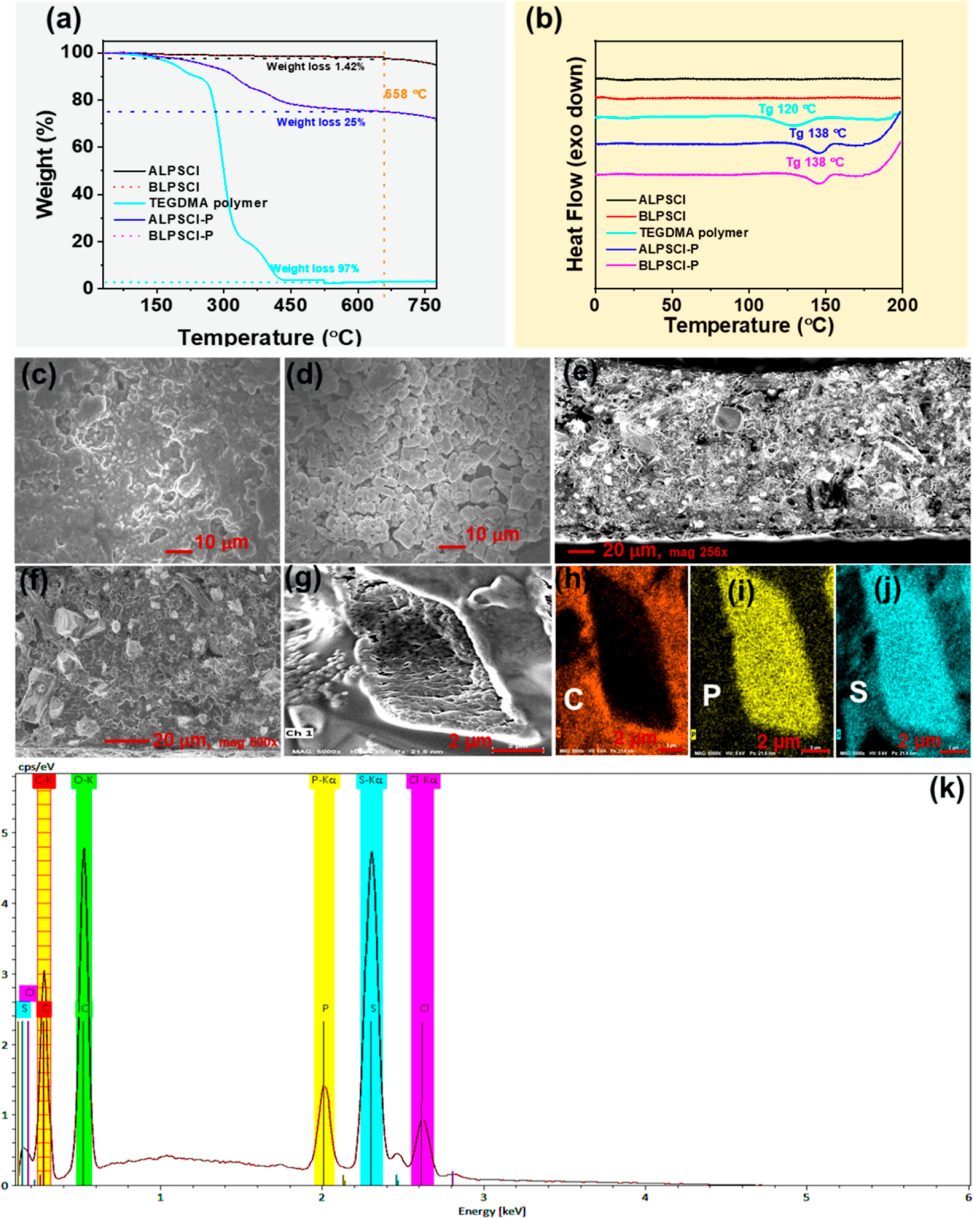
(a) TGA and (b) DSC analyses of prepared electrolytes. SEM images of (c) ALPSCl and (d) BLPSCl electrolytes. Cross-sectional SEM images (e–g) and EDS and elemental mapping (h–k) of BLPSCl–P composite electrolyte.

Thermal stability analysis was conducted on the LPSCl ceramic, cross-linked TEGDMA polymer, ALPSCl–P, and BLPSCl–P electrolytes, as indicated in Figure 2a. Both ALPSCl and BLPSCl ceramics displayed minimal weight loss of approximately 1.42% up to 658 °C and showed ultra-high thermal stability up to 800 °C, which is due to the high crystallinity of the ceramics.^46^ On the other hand, the cross-linked TEGDMA polymer exhibited an initial weight loss of around 70% within the temperature range of 105–336 °C, which could be attributed to the partial decomposition and carbonization of the polymer.^47^ Subsequently, a second weight loss of 97% was observed in the temperature range of 337–440 °C, resulting from the complete decomposition of the polymer. Additionally, both composite membranes, namely, the ALPSCl–P and the BLPSCl–P, displayed a two-step weight loss of ca. 25% between 219 and 658 °C. This weight loss can be attributed to the decomposition of the polymer within the composite electrolytes, indicating the presence of 25 wt % TEGDMA polymer in the composite electrolytes. However, the thermal stability of both composite electrolytes was lower than that of the LPSCl ceramic but higher than most conventional liquid electrolytes.^48^ Therefore, the thermal stability of the as-prepared composite electrolytes is adequate for their practical application in LIB systems.

The *T*_g_ values of the as-prepared electrolytes were investigated by DSC analysis, as shown in Figure 2b. Both ALPSCl and BLPSCl electrolytes showed no obvious exothermic and endothermic behavior up to 200 °C, due to their high thermal stability and crystallinity (Figure S2).^46^ The *T*_g_ values of the TEGDMA polymer and composite electrolytes were ca. 120 and 138 °C, respectively.

The surface morphology, EDS, and elemental analysis of the as-prepared electrolytes were investigated, as depicted in Figures 2c–k and S3. The ceramic electrolyte plays a crucial role in the preparation of highly conductive composite electrolytes. The SEM image illustrates that the BLPSCl ceramic, after ball milling, exhibits reduced aggregation and particle size compared to those of the ALPSCl electrolyte (Figure 2c,d). SEM images of the composite electrolytes, ALPSCl–P and BLPSCl–P, reveal the incorporation of LPSCl particles (Figure S3a,b), which are enveloped by the polymer matrix. Following the in situ polymerization of TEGDMA, the morphology of the LPSCl particles undergoes minimal change, gradually becoming coated by the TEGDMA polymer. This polymer filling within the grain boundaries of the LPSCl particles ensures smooth Li^+^ transport and electronic insulation at these boundaries.^37^ Moreover, the LPSCl ceramic is evenly dispersed throughout the polymer matrix in the BLPSCl–P composite electrolyte and is devoid of any aggregation (Figure 2f). Notably, this uniform distribution of the LPSCl ceramic and polymer facilitates easy movement of Li^+^ in the BLPSCl–P composite electrolyte.^37^ Cross-sectional images, elemental mapping, and EDS analysis of the BLPSCl–P composite electrolyte are presented in Figure 2e–k. It is evident that the TEGDMA polymer forms a layer that envelopes the entire LPSCl particle in the composite electrolyte. This polymer layer can obstruct electronic conduction through the grain boundaries of the LPSCl particles, ultimately safeguarding the solid-state cell from severe self-discharge and dendrite formation.^37^

### 3.3 Ionic Conductivity and Electronic Conductivity of Prepared Electrolytes

**Figure 3:**
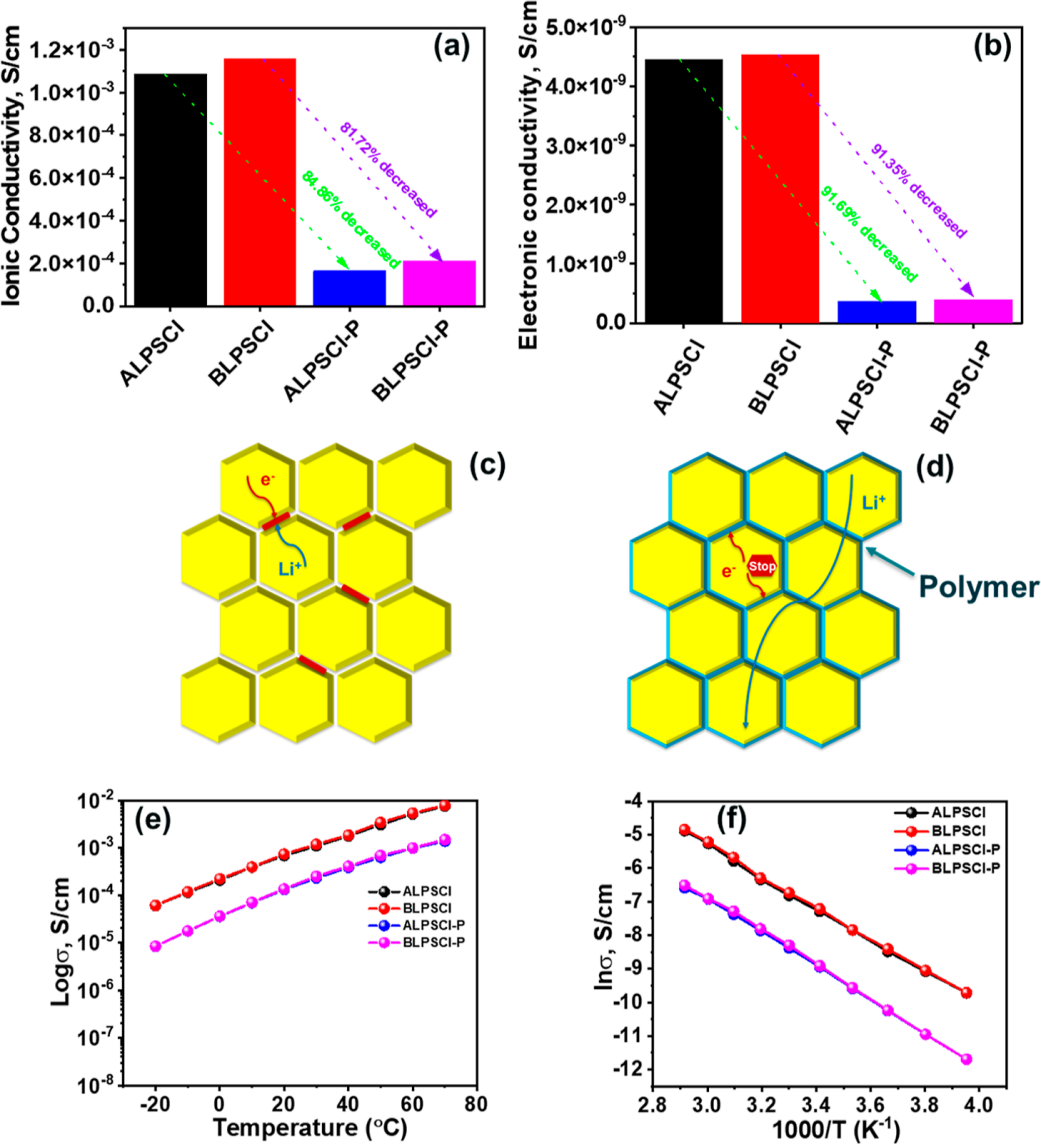
(a) Ionic and (b) electronic conductivities of the prepared SEs at 25 °C. (c) Main challenges of LPSCl electrolyte. (d) Impact of polymer on composite electrolyte. (e) Ionic conductivity vs temperature curves of the prepared SEs. (f) ln(ionic conductivity) vs the inverse of absolute temperatures.

To evaluate σ of the electrolytes, a SS//electrolyte//SS type symmetric cell was prepared under pressure and used AC impedance measurement technique at the temperature range of (−20 to 70) °C. The details of the σ measurement are described in the Supporting Information section and the obtained fitted EIS spectra of the LPSCl and composite electrolytes at 25 °C are shown in Figure S4. The σ value of the ALPSCl ceramic was ca. 1.086 × 10^–3^ S/cm at 25 °C (Figure 3a). While the σ was increased after ball-milling, reaching the value of ca. 1.187 × 10^–3^ S/cm for BLPSCl at 25 °C (Figure 3a), due to the changes in crystallinity, particle size, and aggregation. However, the σ value of ALPSCl–P and BLPSCl–P composite electrolytes were ca. 1.65 × 10^–4^ and 2.21 × 10^–4^ S/cm at 25 °C (Figure 3a), respectively, which are higher or comparable to those of other conventional polymer/ceramic composite electrolytes (Table 1).^3,39,49−54^Figure 3b shows the electronic conductivity of the prepared SEs at 25 °C. The details of the measurement are discussed in the experimental Section 2.2 and also shown in Figures S5 and S6. The electronic conductivities of the SEs, including ALPSCl, BLPSCl, ALPSCl–P, and BLPSCl–P, were ca. 4.45 × 10^–9^, 4.54 × 10^–9^, 3.70 × 10^–10^, and 3.92 × 10^–10^ S/cm, respectively, at 25 °C. The non-negligible electronic conductivities of LPSCl electrolytes lead to smooth electron transport through the LPSCl pellets, resulting in Li-dendrites depositing directly at the grain boundaries and causing serious self-discharge (Figure 3c). However, polymer/LPSCl composite SEs suppress the dendrite growth by reducing electronic conductivity.^37^ By incorporation of polymer into ALPSCl ceramic, the reduction in σ and electronic conductivity for ALPSCl–P was 84.86 and 91.69%, respectively. Similarly, for BLPSCl–P, the reductions were 81.72% for σ and 91.35% for electronic conductivity. These results suggest that the polymer has a greater impact on reducing electronic conductivity compared to σ in the composite electrolytes. The polymer/ceramic composite electrolyte can transport the Li^+^ smoothly while blocking the electron transport at the grain boundary, which helps suppress self-discharge and enhances cycling stability (Figure 3d).^37,39,41^ More importantly, the polymer covered on the surface of LPSCl functions as a protection layer to separate LPSCl and moisture, which improves humidity stability.

**Table 1:** Comparison of the Li^+^ Conductivities at 25 °C of Some Reported Solid Composite Electrolytes with the Prepared Composite Electrolytes^a^

polymer matrix	ceramic	salts	σ, (S/cm)	refs
PEGDME	LPSCl	LiTFSI, LiFSI	4.5 × 10^–^^5^	(39)
PEO	LPSCl	LiTFSI	3.5 × 10^–^^5^	(49)
PEGDME	LPSCl		3.9 × 10^–^^4^	(37)
NBR	LPSCl	LiTFSI	4.0 × 10^–^^4^	(50)
PEGDMA-*co*-LiSTFSI	LiCGC		1.6 × 10^–^^8^	(51)
PL@LCSE	LLZO		1.5 × 10^–^^6^	(52)
PVDF	Li_6.75_La_3_Zr_1.75_Nb_0.25_O_12_	LiClO_4_	9.2 × 10^–^^5^	(53)
poly(dimethylsiloxane)	LATP@PEGDA		2.4 × 10^–^^6^	(54)
PAN	LLTO	LiClO_4_	2.4 × 10^–^^5^	(3)
poly(methyl methacrylate)	Li_6.75_La_3_Zr_1.75_Nb_0.25_O_12_	LiClO_4_	2.2 × 10^–^^5^	(3)
PEO	LLZO	LiTFSI	8.9 × 10^–^^5^	(3)
PEO	LATP		6.8 × 10^–^^6^	(3)
**TEGDMA polymer**	**ALPSCl**		**1.65×10^–^**^**4**^	**this work**
	**BLPSCl**		**2.21×10^–^**^**4**^	

a PEGDME = polyethylene glycol dimethyl ether, NBR = nitrile butadiene rubber, PEGDMA = poly(ethylene glycol) dimethacrylate, LiSTFSI = lithium 4-styrenesulfonyl-(trifluoromethylsulfonyl)imide, LiCGC = lithium-ion-conducting glass ceramic powder, PL@LCSE = PEO + Ta-doped garnet Li_6.4_La_3_Zr_1.4_Ta_0.6_O_12_ + lithium 4-styrenesulfonyl-(trifluoromethylsulfonyl)imide, and LATP@PEGDA = Li_1.3_Al_0.3_Ti_1.7_(PO_4_)_3_ particles + poly(ethylene glycol) diacrylate.

The σ values of ALPSCl and BLPSCl ceramics were ca. 5.99 × 10^–5^ and 6.04 × 10^–5^ S/cm at −20 °C. While the σ was gradually raised with increasing temperature (Figure 3e), reaching the value of ca. 7.57 × 10^–3^ and 7.87 × 10^–3^ S/cm at 70 °C. Accordingly, the σ values of the composite electrolytes were ca. 8.34 × 10^–6^ S/cm and 8.36 × 10^–6^ S/cm for ALPSCl–P and BLPSCl–P, respectively, at −20 °C. While with the increase of temperature, both of the composite electrolytes exhibited higher σ, ALPSCl–P and BLPSCl–P, showed enhanced σ values of ca. 1.37 × 10^–3^ and 1.49 × 10^–3^ S/cm, respectively, at 70 °C (Figure 3e). Additionally, in order to investigate the temperature dependency of the electrolytes’ σ, we created a graph by plotting the ln σ against the reciprocal of absolute temperatures, as illustrated in Figure 3f. This graph displayed a linear correlation between ln σ and temperature, closely resembling the typical Arrhenius plot. This analysis yielded activation energy (*E*_a_) values of approximately 0.21, 0.20, 0.25, and 0.23 eV for the ALPSCl, BLPSCl, ALPSCl–P, and BLPSCl–P electrolytes, respectively. The relatively low *E*_a_ values for these electrolytes are in line with the observed high σ.

### 3.4 Compatibility of Prepared Electrolytes with Li-Metal

**Figure 4:**
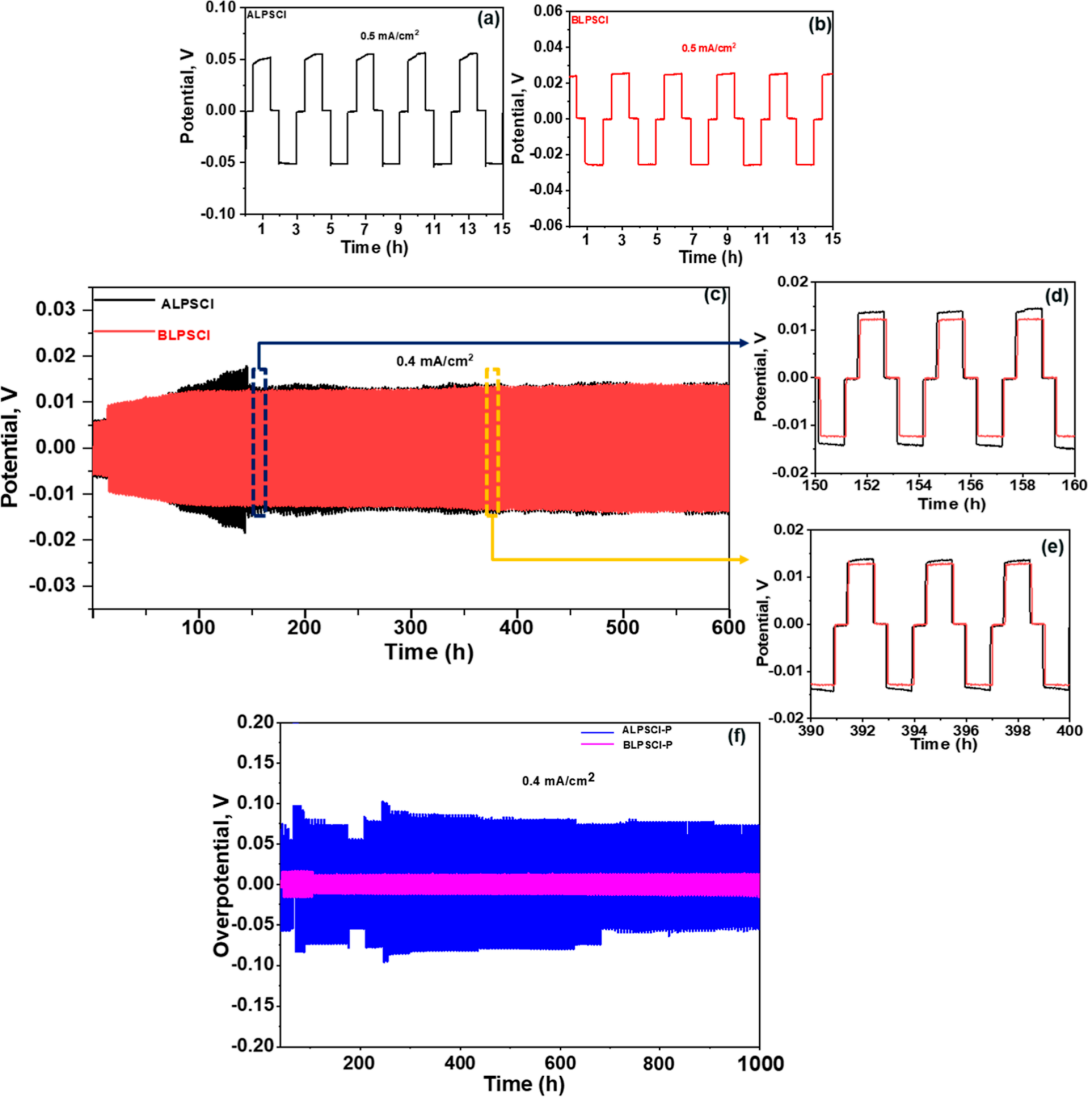
(a,b) Li plating-striping curves at 0.5 mA/cm^2^ current density for ALPSCl and BLPSCl electrolytes. (c–e) Li plating-striping curves at 0.4 mA/cm^2^ current density for ALPSCl and BLPSCl electrolytes. (f) Li plating-striping curves at 0.4 mA/cm^2^ current density for ALPSCl–P and BLPSCl–P electrolytes.

Figure 4a–f illustrates the cycling stability of Li-metal symmetric cells using ALPSCl and BLPSCl ceramic electrolytes at 25 °C. The experimental details can be found in the Supporting Information section (Figure S7). Both cells, with ALPSCl and BLPSCl electrolytes, showed different behaviors at 0.5 mA/cm^2^ current density (Figure 4a,b). Notably, the cell with ALPSCl electrolyte displayed wedge-shaped voltage plateaus at a current density of 0.5 mA/cm^2^ due to the increasing Li^+^ transport resistance during lithium deposition, leading to uneven lithium plating (Figure 4a). This uneven plating/stripping, along with the solid-solid point contact and volume changes in the Li-metal anode, resulted in a continuous decrease in the effective contact area between the Li-metal anode and LPSCl electrolyte.^55^ Consequently, the limited contact area contributed to higher local current density and exacerbated the uneven deposition of lithium metal, thereby promoting dendrite growth in the SE.^55^ Conversely, the cell with BLPSCl electrolyte exhibited a potential curve indicating a uniform current distribution on the BLPSCl electrolyte at a current density of 0.5 mA/cm^2^ (Figure 4b). Figure 4c–e shows the voltage–time profile of ALPSCl and BLPSCl electrolytes at 0.4 mA/cm^2^ at 25 °C. The cells with ALPSCl and BLPSCl electrolytes exhibited low cycling stability up to 600 h (225 cycles) and this low cycling stability is responsible for the Li deposition in the bulk LPSCl, reduction of Li^+^ at the grain boundaries of the LPSCl electrolyte.^37^ However, compared to ALPSCl, the BLPSCl electrolyte showed better, uniform, smooth, and dendrite-free Li-deposition. The improved performance of the ball-milled electrolyte can be attributed to its distinct morphology, smaller particle size, and reduced aggregation compared to the ALPSCl electrolyte. The effect of a polymer on the suppression of Li dendrites was also investigated (Figure 4f). The cells containing polymer/ceramic electrolytes demonstrated improved Li plating/stripping cycling performance, allowing them to operate for up to 1000 h (375 cycles) at 0.4 mA/cm^2^. However, the disparity in cycling stability between the composite electrolytes and LPSCl electrolytes can be attributed to different Li deposition models. The grain boundary of the LPSCl particle serves as a pathway for Li deposition, facilitating easy electron transfer between adjacent LPSCl particles without any barriers.^37^ Consequently, continuous Li deposition and the growth of Li dendrites along the grain boundaries ultimately lead to a short circuit.^37^ In contrast, the incorporation of the TEGDMA polymer in the LPSCl ceramic electrolyte obstructs electron transport at the grain boundaries,^37^ resulting in suppressed Li dendrite growth in the bulk LPSCl and improved cycle life for Li–Li symmetric cells. The TEGDMA polymer shields the grain boundary of the LPSCl ceramics, impeding the movement of electrons between LPSCl particles.^37^ However, the electrochemical performance of Li–Li symmetric cells with LPSCl/polymer composite electrolytes demonstrates excellent Li plating-stripping performance, surpassing or matching that of other SSBs (Table S1). In addition, incorporating the polymer led to an increase in overpotential due to the reduced σ, which can be detrimental to high-rate capability.

As shown in Figure S8a,b, the cell resistance of the Li–Li symmetric cells with ALPSCl and BLPSCl electrolytes was increased for the first few cycles and then stabilized, which can be attributed to the interfacial reactions between Li and LPSCl and interphase formation.^37,56−58^ The corresponding EIS results during cycling of the ALPSCl–P and BLPSCl–P composite electrolyte confirmed the high stability and Li dendrite-free behavior. As shown in Figure S8c,d, the slight increase in cell resistance during the first few cycles reflected the SEI formation process at the Li/SE interface, but the stabilized resistance after the first few cycles supported the stable Li plating/stripping behavior. Moreover, the cell utilizing BLPSCl–P composite electrolyte exhibited superior characteristics, such as lower over potential and smoother Li deposition behavior, compared to the ALPSCl–P composite electrolyte. These improvements can be attributed to the reduced particle size and altered morphology resulting from the ball milling of the LPSCl electrolyte. These findings align perfectly with the results obtained from σ, electronic conductivity, XRD, XPS, and SEM analyses. The huge difference in Li plating-stripping behavior with and without polymer further highlights the positive effect of the polymer on suppressing Li dendrite growth.

### 3.5 Battery Performances of Prepared Electrolytes

**Figure 5:**
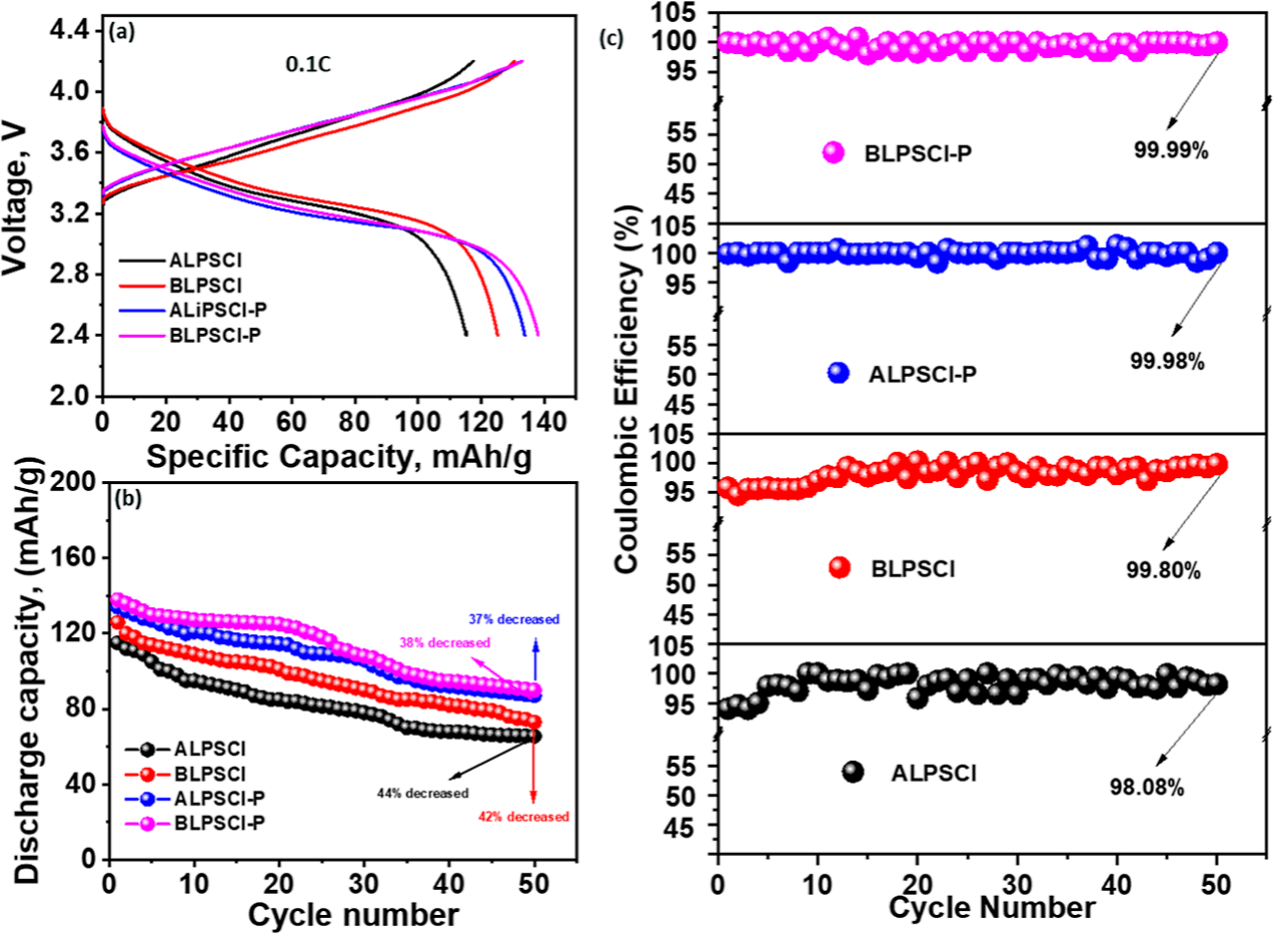
(a) CD plots of the as-prepared electrolyte solutions based on the coated NMC811//SE//Li–In cell at 0.1C rate. (b) Specific discharge capacity plots of the cells as a function of the CD cycles at 0.1C. (c) Coulombic efficiency of the cells with as-prepared SEs as a function of the CD cycles.

The battery performance of the prepared electrolytes was examined using an all-solid-state cell that had a coated NMC811//electrolyte//Li–In configuration at a temperature of 25 °C. The choice of coated NMC811 as the cathode material was based on its high energy density, cycling performance, and theoretical capacity (180 mA h/g at 0.1C).^6,59^ The cells’ CD plots were measured by applying constant currents (0.1, 0.2, 0.3, and 0.5C) across the potential range of 2.4–4.2 V. The C rate was determined using the weight of the active cathode material (12 mg). Figure 5a illustrates the CD plots of the cells at 0.1C, up to a potential of 4.2 V, while Figure 5b,c depicts the changes in discharge specific capacity (*C*_sp_) and Coulombic efficiency as the number of CD cycles increases at 0.1C. At a rate of 0.1C, the *C*_sp_ values of the solid-state cells with ALPSCl, BLPSCl, ALPSCl–P, and BLPSCl–P electrolytes were approximately 115, 125, 134, and 138 mA h/g, respectively (Figure 5a). These values are comparable to or higher than those reported for other SEs (Table S1). Figure S9a,b depicts the EIS curves for the all-solid-state lithium cells prepared with four different SEs, both before cycling and after 50 cycles at 0.1C. It is observed that the cell resistance for all four cells experienced a slight increase. This increase can be attributed to interfacial reactions between lithium and LPSCl, along with the formation of an interphase.^37,56^ Furthermore, after 50 CD cycles, the LIB with these electrolytes exhibited *C*_sp_ values of around 65, 73, 87, and 90 mA h/g for ALPSCl, BLPSCl, ALPSCl–P, and BLPSCl–P electrolytes, respectively, at 0.1C. The capacity was decreased by approximately 44, 42, 37, and 38% of the initial *C*_sp_ (Figure 5b). Additionally, the BLPSCl–P electrolyte demonstrated excellent cycling and electrochemical stability, showing promise for the development of high-voltage ASSLIBs. Additionally, the rate capability of the cells was investigated at various current densities from 0.1 to 0.5C, as shown in Figure S10. The cell with BLPSCl–P electrolyte delivered a capacity of over 59 mA h/g at a high current density of 0.5C, and no short circuit was observed. The Coulombic efficiencies of the cell using ALPSCl, BLPSCl, ALPSCl–P, and BLPSCl–P electrolytes were approximately 94.05, 95.82, 99.85, and 99.97%, respectively, during the first CD cycle (Figure 5c). These efficiencies significantly improved to approximately 98.08, 99.80, 99.98, and 99.99%, respectively, after 50 CD cycles owing to the enhanced interfacial contact between the electrode and electrolyte.^60,61^ More importantly, both at the initial and following 50 cycles, the cells utilizing composite electrolytes, specifically ALPSCL–P and BLPSCL–P, demonstrated higher Coulombic efficiencies compared with cells employing individual ALPSCl and BLPSCL electrolytes. Additionally, the fluctuation in Coulombic efficiency observed in cells with ALPSCl and BLPSCl electrolytes is higher than that in cells with ALPSCl–P and BLPSCl–P composite electrolytes. This phenomenon is indeed common in solid-state batteries with LPSCl electrolytes.^44,57^ These observations strongly suggest that the composite electrolytes exhibit substantial electrochemical stability and enduring cycling performance within a potential range of up to 4.2 V.

## 4 Conclusions

We synthesized composite electrolytes based on LPSCl ceramic and polymer to suppress Li dendrite growth and self--discharge in SSBs. We utilized the TEGDMA monomer for in situ polymerization via thermal curing to prepare the composite electrolyte. Additionally, we employed ball-milling to modify the LPSCl ceramic’s particle size and morphology. At 25 °C, the BLPSCl–P electrolyte exhibited slightly higher σ of 2.12 × 10^–4^ S/cm compared to the ALPSCl–P composite electrolyte’s σ of 1.65 × 10^–4^ S/cm. The electronic insulating properties of the TEGDMA polymer reduced the overall electronic conductivity of the composite electrolyte, effectively inhibiting the reduction of Li^+^ by electrons to Li-metal at the grain boundaries. Consequently, both composite electrolytes demonstrated excellent compatibility with Li-metal, maintaining stable cycling for 1000 h (375 cycles), in contrast to ALPSCl and BLPSCl electrolytes, which remained stable for only 600 h (225 cycles) at a current density of 0.4 mA/cm^2^. Furthermore, the SSB using the BLPSCl–P composite electrolyte achieved a high *C*_sp_ of 138 mA h/g, initial Coulombic efficiency of 99.97%, and good capacity retention at 0.1C and 25 °C. Apart from enhancing Li dendrite suppression and self-discharge mitigation, the TEGDMA polymer coating also shielded LPSCl from moisture, thereby improving humidity stability. To enhance the electrochemical performance of these composite electrolytes, our ongoing research in the laboratory is centered on developing cathode materials and compositions with well-matched energy band positions. Additionally, our research delves into the electrolyte–electrode interface and thoroughly investigates dendrite growth using optical microscopic images. We believe that our technique and composite electrolytes hold great promise for the advancement of practical ASSLBs with enhanced safety and stability.

## Supporting Information Available

XPS spectra, SEM image, charge–discharge plot, EIS spectra, XRD spectra, electronic conductivity, ionic conductivity, and specific discharge capacity and Li-Li cycling stability of some recently reported composite SEs for ASSLBs (PDF)

## References

[ref1] Liu X.; Xiao Z.; Peng H.; Jiang D.; Xie H.; Sun Y.; Zhong S.; Qian Z.; Wang R. Rational Design of LLZO/Polymer Solid Electrolytes for Solid-State Batteries. Chem.–Asian J. 2022, 17 (24), e202200929 10.1002/asia.202200929. 36210332

[ref2] Lau J.; DeBlock R. H.; Butts D. M.; Ashby D. S.; Choi C. S.; Dunn B. S. Sulfide Solid Electrolytes for Lithium Battery Applications. Adv. Energy Mater. 2018, 8 (27), 1800933 10.1002/aenm.201800933.

[ref3] Li S.; Zhang S. Q.; Shen L.; Liu Q.; Ma J. B.; Lv W.; He Y. B.; Yang Q. H. Progress and Perspective of Ceramic/Polymer Composite Solid Electrolytes for Lithium Batteries. Adv. Sci. 2020, 7 (5), 1903088 10.1002/advs.201903088. PMC7055568 32154083

[ref4] Yu X.; Manthiram A. A Review of Composite Polymer-Ceramic Electrolytes for Lithium Batteries. Energy Storage Mater. 2021, 34 (10), 282–300. 10.1016/j.ensm.2020.10.006.

[ref5] Liu K.; Wu M.; Wei L.; Lin Y.; Zhao T. A Composite Solid Electrolyte with a Framework of Vertically Aligned Perovskite for All-Solid-State Li-Metal Batteries. J. Membr. Sci. 2020, 610 (05), 118265 10.1016/j.memsci.2020.118265.

[ref6] Wu J.; Shen L.; Zhang Z.; Liu G.; Wang Z.; Zhou D.; Wan H.; Xu X.; Yao X. All-Solid-State Lithium Batteries with Sulfide Electrolytes and Oxide Cathodes. Electrochem. Energy Rev. 2021, 4 (1), 101–135. 10.1007/s41918-020-00081-4.

[ref7] He L.; Oh J. A. S.; Watarai K.; Morita M.; Zhao Y.; Sun Q.; Sakamoto T.; Lu L.; Adams S. Electromechanical Failure of NASICON-Type Solid-State Electrolyte-Based All-Solid-State Li-Ion Batteries. Chem. Mater. 2021, 33 (17), 6841–6852. 10.1021/acs.chemmater.1c01564.

[ref8] Tao X.; Liu Y.; Liu W.; Zhou G.; Zhao J.; Lin D.; Zu C.; Sheng O.; Zhang W.; Lee H. W.; Cui Y. Solid-State Lithium-Sulfur Batteries Operated at 37 °C with Composites of Nanostructured Li_7_La_3_Zr_2_O_12_/Carbon Foam and Polymer. Nano Lett. 2017, 17 (5), 2967–2972. 10.1021/acs.nanolett.7b00221. 28388080

[ref9] Yan C.; Zhu P.; Jia H.; Du Z.; Zhu J.; Orenstein R.; Cheng H.; Wu N.; Dirican M.; Zhang X. Garnet-Rich Composite Solid Electrolytes for Dendrite-Free, High-Rate, Solid-State Lithium-Metal Batteries. Energy Storage Mater. 2020, 26 (08), 448–456. 10.1016/j.ensm.2019.11.018.

[ref10] Yu X.; Liu Y.; Goodenough J. B.; Manthiram A. Rationally Designed PEGDA-LLZTO Composite Electrolyte for Solid-State Lithium Batteries. ACS Appl. Mater. Interfaces 2021, 13 (26), 30703–30711. 10.1021/acsami.1c07547. 34180236

[ref11] Gao H.; Huang Y.; Zhang Z.; Huang J.; Li C. Li6.7La3Zr1.7Ta0.15Nb0.15O12 Enhanced UV-Cured Poly(Ethylene Oxide)-Based Composite Gel Polymer Electrolytes for Lithium Metal Batteries. Electrochim. Acta 2020, 360, 137014 10.1016/j.electacta.2020.137014.

[ref12] Ao X.; Wang X.; Tan J.; Zhang S.; Su C.; Dong L.; Tang M.; Wang Z.; Tian B.; Wang H. Nanocomposite with Fast Li^+^ Conducting Percolation Network: Solid Polymer Electrolyte with Li^+^ Non-Conducting Filler. Nano Energy 2021, 79 (10), 105475 10.1016/j.nanoen.2020.105475.

[ref13] Jia M.; Zhao N.; Bi Z.; Fu Z.; Xu F.; Shi C.; Guo X. Polydopamine-Coated Garnet Particles Homogeneously Distributed in Poly(Propylene Carbonate) for the Conductive and Stable Membrane Electrolytes of Solid Lithium Batteries. ACS Appl. Mater. Interfaces 2020, 12 (41), 46162–46169. 10.1021/acsami.0c13434. 32935964

[ref14] Xu T.; Chen C.; Jin T.; Lou S.; Zhang R.; Xiao X.; Huang X.; Yang Y. Chemical Heterogeneity in PAN/LLZTO Composite Electrolytes by Synchrotron Imaging. J. Electrochem. Soc. 2021, 168 (11), 110522 10.1149/1945-7111/ac352a.

[ref15] Bag S.; Zhou C.; Kim P. J.; Pol V. G.; Thangadurai V. LiF Modified Stable Flexible PVDF-Garnet Hybrid Electrolyte for High-Performance All-Solid-State Li-S Batteries. Energy Storage Mater. 2020, 24 (8), 198–207. 10.1016/j.ensm.2019.08.019.

[ref16] Itoh M.; Inaguma Y.; Jung W.-H.; Chen L.; Nakamura T. High Lithium Ion Conductivity in the Perovskite-Type Compounds Ln_12_Li_12_TiO_3_(Ln = La, Pr, Nd, Sm). Solid State Ionics 1994, 70–71 (1), 203–207. 10.1016/0167-2738(94)90310-7.

[ref17] Mercier R.; Malugani J.-P.; Fahys B.; Robert G. Superionic Conduction in Li_2_S-P_2_S_5_-LiI-Glasses. Solid State Ionics 1981, 5, 663–666. 10.1016/0167-2738(81)90341-6.

[ref18] Trevey J. E.; Jung Y. S.; Lee S. H. High Lithium Ion Conducting Li_2_S-GeS_2_-P_2_S_5_ Glass-Ceramic Solid Electrolyte with Sulfur Additive for All Solid-State Lithium Secondary Batteries. Electrochim. Acta 2011, 56 (11), 4243–4247. 10.1016/j.electacta.2011.01.086.

[ref19] Sahu G.; Lin Z.; Li J.; Liu Z.; Dudney N.; Liang C. Air-Stable, High-Conduction Solid Electrolytes of Arsenic-Substituted Li_4_SnS_4_. Energy Environ. Sci. 2014, 7 (3), 1053–1058. 10.1039/C3EE43357A.

[ref20] Wenzel S.; Weber D. A.; Leichtweiss T.; Busche M. R.; Sann J.; Janek J. Interphase Formation and Degradation of Charge Transfer Kinetics between a Lithium Metal Anode and Highly Crystalline Li_7_P_3_S_11_ Solid Electrolyte. Solid State Ionics 2016, 286, 24–33. 10.1016/j.ssi.2015.11.034.

[ref21] Kuhn A.; Gerbig O.; Zhu C.; Falkenberg F.; Maier J.; Lotsch B. V. A New Ultrafast Superionic Li-Conductor: Ion Dynamics in Li_11_Si_2_PS_12_ and Comparison with Other Tetragonal LGPS-Type Electrolytes. Phys. Chem. Chem. Phys. 2014, 16 (28), 14669–14674. 10.1039/C4CP02046D. 24916653

[ref22] Ye L.; Li X. A Dynamic Stability Design Strategy for Lithium Metal Solid State Batteries. Nature 2021, 593 (7858), 218–222. 10.1038/s41586-021-03486-3. 33981053

[ref23] Wang C.; Adair K. R.; Liang J.; Li X.; Sun Y.; Li X.; Wang J.; Sun Q.; Zhao F.; Lin X.; Li R.; Huang H.; Zhang L.; Yang R.; Lu S.; Sun X. Solid-State Plastic Crystal Electrolytes: Effective Protection Interlayers for Sulfide-Based All-Solid-State Lithium Metal Batteries. Adv. Funct. Mater. 2019, 29 (26), 1900392 10.1002/adfm.201900392.

[ref24] Zhang Z.; Chen S.; Yang J.; Wang J.; Yao L.; Yao X.; Cui P.; Xu X. Interface Re-Engineering of Li_10_GeP_2_S_12_ Electrolyte and Lithium Anode for All-Solid-State Lithium Batteries with Ultralong Cycle Life. ACS Appl. Mater. Interfaces 2018, 10 (3), 2556–2565. 10.1021/acsami.7b16176. 29278487

[ref25] Yu C.; Zhao F.; Luo J.; Zhang L.; Sun X. Recent Development of Lithium Argyrodite Solid-State Electrolytes for Solid-State Batteries: Synthesis, Structure, Stability and Dynamics. Nano Energy 2021, 83 (1), 105858 10.1016/j.nanoen.2021.105858.

[ref26] Jung W. D.; Kim J. S.; Choi S.; Kim S.; Jeon M.; Jung H. G.; Chung K. Y.; Lee J. H.; Kim B. K.; Lee J. H.; Kim H. Superionic Halogen-Rich Li-Argyrodites Using in Situ Nanocrystal Nucleation and Rapid Crystal Growth. Nano Lett. 2020, 20, 2303–2309. 10.1021/acs.nanolett.9b04597. 32150419

[ref27] Wenzel S.; Randau S.; Leichtweiß T.; Weber D. A.; Sann J.; Zeier W. G.; Janek J. Direct Observation of the Interfacial Instability of the Fast Ionic Conductor Li_10_GeP_2_S_12_ at the Lithium Metal Anode. Chem. Mater. 2016, 28 (7), 2400–2407. 10.1021/acs.chemmater.6b00610.

[ref28] Singh D. K.; Henss A.; Mogwitz B.; Gautam A.; Horn J.; Krauskopf T.; Burkhardt S.; Sann J.; Richter F. H.; Janek J. Li_6_PS_5_Cl Microstructure and Influence on Dendrite Growth in Solid-State Batteries with Lithium Metal Anode. Cell Rep. Phys. Sci. 2022, 3 (9), 101043 10.1016/j.xcrp.2022.101043.

[ref29] Lee S. E.; Sim H. T.; Lee Y. J.; Hong S. B.; Chung K. Y.; Jung H. G.; Kim D. W. Li_6_PS_5_Cl-Based Composite Electrolyte Reinforced with High-Strength Polyester Fibers for All-Solid-State Lithium Batteries. J. Power Sources 2022, 542 (6), 231777 10.1016/j.jpowsour.2022.231777.

[ref30] Zou C.; Yang L.; Luo K.; Liu L.; Tao X.; Yi L.; Liu X.; Luo Z.; Wang X. Ionic Conductivity and Interfacial Stability of Li6PS5Cl-Li6.5La3Zr1.5Ta0.5O12 Composite Electrolyte. J. Solid State Electrochem. 2021, 25 (10–11), 2513–2525. 10.1007/s10008-021-05004-x.

[ref31] Lee Y. G.; Fujiki S.; Jung C.; Suzuki N.; Yashiro N.; Omoda R.; Ko D. S.; Shiratsuchi T.; Sugimoto T.; Ryu S.; Ku J. H.; Watanabe T.; Park Y.; Aihara Y.; Im D.; Han I. T. High-Energy Long-Cycling All-Solid-State Lithium Metal Batteries Enabled by Silver-Carbon Composite Anodes. Nat. Energy 2020, 5 (4), 299–308. 10.1038/s41560-020-0575-z.

[ref32] Wan H.; Wang Z.; Liu S.; Zhang B.; He X.; Zhang W.; Wang C. Critical Interphase Overpotential as a Lithium Dendrite-Suppression Criterion for All-Solid-State Lithium Battery Design. Nat. Energy 2023, 8 (5), 473–481. 10.1038/s41560-023-01231-w.

[ref33] Wu J.; Liu S.; Han F.; Yao X.; Wang C. Lithium/Sulfide All-Solid-State Batteries Using Sulfide Electrolytes. Adv. Mater. 2021, 33 (6), 2000751 10.1002/adma.202000751. 32812301

[ref34] Liu G.; Weng W.; Zhang Z.; Wu L.; Yang J.; Yao X. Densified Li_6_PS_5_Cl Nanorods with High Ionic Conductivity and Improved Critical Current Density for All-Solid-State Lithium Batteries. Nano Lett. 2020, 20 (9), 6660–6665. 10.1021/acs.nanolett.0c02489. 32787073

[ref35] Guo R.; Zhang K.; Zhao W.; Hu Z.; Li S.; Zhong Y.; Yang R.; Wang X.; Wang J.; Wu C.; Bai Y. Interfacial Challenges and Strategies toward Practical Sulfide-Based Solid-State Lithium Batteries. Energy Mater. Adv. 2023, 4, 1–31. 10.34133/energymatadv.0022.

[ref36] Reddy M. V.; Julien C. M.; Mauger A.; Zaghib K. Sulfide and Oxide Inorganic Solid Electrolytes for All-Solid-State Li Batteries: A Review. Nanomaterials 2020, 10 (8), 1606 10.3390/nano10081606. 32824170 PMC7466729

[ref37] Yang X.; Gao X.; Jiang M.; Luo J.; Yan J.; Fu J.; Duan H.; Zhao S.; Tang Y.; Yang R.; Li R.; Wang J.; Huang H.; Veer Singh C.; Sun X. Grain Boundary Electronic Insulation for High-Performance All-Solid-State Lithium Batteries. Angew. Chem., Int. Ed. 2023, 62 (5), e202215680 10.1002/anie.202215680. 36446742

[ref38] Zou C.; Yang L.; Luo K.; Liu L.; Tao X.; Yi L.; Liu X.; Luo Z.; Wang X. Preparation and Performances of Poly (Ethylene Oxide)-Li_6_PS_5_Cl Composite Polymer Electrolyte for All-Solid-State Lithium Batteries. J. Electroanal. Chem. 2021, 900 (6), 115739 10.1016/j.jelechem.2021.115739.

[ref39] Huo H.; Jiang M.; Mogwitz B.; Sann J.; Yusim Y.; Zuo T. T.; Moryson Y.; Minnmann P.; Richter F. H.; Veer Singh C.; Janek J. Interface Design Enabling Stable Polymer/Thiophosphate Electrolyte Separators for Dendrite-Free Lithium Metal Batteries. Angew. Chem., Int. Ed. 2023, 62 (14), e202218044 10.1002/anie.202218044. 36646631

[ref40] Khomein P.; Byeon Y. W.; Liu D.; Yu J.; Minor A. M.; Kim H.; Liu G. Lithium Phosphorus Sulfide Chloride-Polymer Composite via the Solution-Precipitation Process for Improving Stability toward Dendrite Formation of Li-Ion Solid Electrolyte. ACS Appl. Mater. Interfaces 2023, 15 (9), 11723–11730. 10.1021/acsami.2c21302. 36827520 PMC9999344

[ref41] Wang S.; Zhang X.; Liu S.; Xin C.; Xue C.; Richter F.; Li L.; Fan L.; Lin Y.; Shen Y.; Janek J.; Nan C. W. High-Conductivity Free-Standing Li_6_PS_5_Cl/Poly(Vinylidene Difluoride) Composite Solid Electrolyte Membranes for Lithium-Ion Batteries. J. Mater. 2020, 6 (1), 70–76. 10.1016/j.jmat.2019.12.010.

[ref42] Pavia D. L.; Lampman G. M.; Kriz G. S.; Vyvyan J. A. Introduction to Spectroscopy, 4th ed.; Brookescole Publishers: CA, 2008.

[ref43] Chen Y. T.; Marple M. A. T.; Tan D. H. S.; Ham S. Y.; Sayahpour B.; Li W. K.; Yang H.; Lee J. B.; Hah H. J.; Wu E. A.; Doux J. M.; Jang J.; Ridley P.; Cronk A.; Deysher G.; Chen Z.; Meng Y. S. Investigating Dry Room Compatibility of Sulfide Solid-State Electrolytes for Scalable Manufacturing. J. Mater. Chem. A 2022, 10 (13), 7155–7164. 10.1039/D1TA09846B.

[ref44] Auvergniot J.; Cassel A.; Ledeuil J. B.; Viallet V.; Seznec V.; Dedryvère R. Interface Stability of Argyrodite Li_6_PS_5_Cl toward LiCoO_2_, LiNi_1/3_Co_1/3_Mn_1/3_O_2_, and LiMn_2_O_4_ in Bulk All-Solid-State Batteries. Chem. Mater. 2017, 29 (9), 3883–3890. 10.1021/acs.chemmater.6b04990.

[ref45] Rajagopal R.; Subramanian Y.; Jung Y. J.; Kang S.; Ryu K. S. Rapid Synthesis of Highly Conductive Li6PS5Cl Argyrodite-Type Solid Electrolytes Using Pyridine Solvent. ACS Appl. Energy Mater. 2022, 5 (8), 9266–9272. 10.1021/acsaem.2c01157.

[ref46] Wang S.; Wu Y.; Li H.; Chen L.; Wu F. Improving Thermal Stability of Sulfide Solid Electrolytes: An Intrinsic Theoretical Paradigm. InfoMat. 2022, 4 (8), e12316 10.1002/inf2.12316.

[ref47] Cavalheiro É. T. G.; Bannach G.; Cavalheiro C. C.; Calixto L. THERMOANALYTICAL STUDY OF MONOMERS: BisGMA, BisEMA, TEGDMA, UDMA. Braz. J. Therm. Anal. 2015, 4 (1–2), 28 10.18362/bjta.v4.i1-2.81.

[ref48] Ahmed F.; Rahman M. M.; Chandra Sutradhar S.; Siraj Lopa N.; Ryu T.; Yoon S.; Choi I.; Kim J.; Jin Y.; Kim W. Synthesis of an Imidazolium Functionalized Imide Based Electrolyte Salt and Its Electrochemical Performance Enhancement with Additives in Li-Ion Batteries. J. Ind. Eng. Chem. 2019, 78, 178–185. 10.1016/j.jiec.2019.06.016.

[ref49] Simon F. J.; Hanauer M.; Richter F. H.; Janek J. Interphase Formation of PEO20:LiTFSI-Li_6_PS_5_Cl Composite Electrolytes with Lithium Metal. ACS Appl. Mater. Interfaces 2020, 12 (10), 11713–11723. 10.1021/acsami.9b22968. 32052956

[ref50] Xia Y.; Li J.; Xiao Z.; Zhou X.; Zhang J.; Huang H.; Gan Y.; He X.; Zhang W. Argyrodite Solid Electrolyte-Integrated Ni-Rich Oxide Cathode with Enhanced Interfacial Compatibility for All-Solid-State Lithium Batteries. ACS Appl. Mater. Interfaces 2022, 14, 33361–33369. 10.1021/acsami.2c08940. 35834669

[ref51] Merrill L. C.; Chen X. C.; Zhang Y.; Ford H. O.; Lou K.; Zhang Y.; Yang G.; Wang Y.; Wang Y.; Schaefer J. L.; Dudney N. J. Polymer-Ceramic Composite Electrolytes for Lithium Batteries: A Comparison between the Single-Ion-Conducting Polymer Matrix and Its Counterpart. ACS Appl. Energy Mater. 2020, 3 (9), 8871–8881. 10.1021/acsaem.0c01358.

[ref52] Liu M.; Guan X.; Liu H.; Ma X.; Wu Q.; Ge S.; Zhang H.; Xu J. Composite Solid Electrolytes Containing Single-Ion Lithium Polymer Grafted Garnet for Dendrite-Free, Long-Life All-Solid-State Lithium Metal Batteries. Chem. Eng. J. 2022, 445, 136436 10.1016/j.cej.2022.136436.

[ref53] Li R.; Wu D.; Yu L.; Mei Y.; Wang L.; Li H.; Hu X. Unitized Configuration Design of Thermally Stable Composite Polymer Electrolyte for Lithium Batteries Capable of Working Over a Wide Range of Temperatures. Adv. Eng. Mater. 2019, 21 (7), 1–9. 10.1002/adem.201900055.

[ref54] Liu X.; Peng S.; Gao S.; Cao Y.; You Q.; Zhou L.; Jin Y.; Liu Z.; Liu J. Electric-Field-Directed Parallel Alignment Architecting 3D Lithium-Ion Pathways within Solid Composite Electrolyte. ACS Appl. Mater. Interfaces 2018, 10 (18), 15691–15696. 10.1021/acsami.8b01631. 29667402

[ref55] Yu P.; Ye Y.; Zhu J.; Xia W.; Zhao Y. Optimized Interfaces in Anti-Perovskite Electrolyte-Based Solid-State Lithium Metal Batteries for Enhanced Performance. Front. Chem. 2021, 9 (12), 1–9. 10.3389/fchem.2021.786956. PMC8733680 35004611

[ref56] Pathak R.; Chen K.; Gurung A.; Reza K. M.; Bahrami B.; Pokharel J.; Baniya A.; He W.; Wu F.; Zhou Y.; Xu K.; Qiao Q. Q. Fluorinated Hybrid Solid-Electrolyte-Interphase for Dendrite-Free Lithium Deposition. Nat. Commun. 2020, 11 (1), 93 10.1038/s41467-019-13774-2. 31900398 PMC6941966

[ref57] Zou C.; Yang L.; Luo K.; Liu L.; Tao X.; Yi L.; Liu X.; Zhang X.; Wang X. In Situ Formed Protective Layer: Toward a More Stable Interface between the Lithium Metal Anode and Li_6_PS_5_Cl Solid Electrolyte. ACS Appl. Energy Mater. 2022, 5 (7), 8428–8436. 10.1021/acsaem.2c00971.

[ref58] Zuo T.-T.; Walther F.; Teo J. H.; Rueß R.; Wang Y.; Rohnke M.; Schröder D.; Nazar L. F.; Janek J. Impact of the Chlorination of Lithium Argyrodites on the Electrolyte/Cathode Interface in Solid-State Batteries. Angew. Chem., Int. Ed. 2023, 62 (7), e202213228 10.1002/anie.202213228. PMC10107527 36416271

[ref59] Liu H.; Xie Z.; Qu W.; Dy E.; Niketic S.; Brueckner S.; Tsay K.; Fuller E.; Bock C.; Zaker N.; Botton G. A. High-Voltage Induced Surface and Intragranular Structural Evolution of Ni-Rich Layered Cathode. Small 2022, 18 (19), 2200627 10.1002/smll.202200627. 35411712

[ref60] Ahmed F.; Choi I.; Rahman M. M.; Jang H.; Ryu T.; Yoon S.; Jin L.; Jin Y.; Kim W. Remarkable Conductivity of a Self-Healing Single-Ion Conducting Polymer Electrolyte, Poly(Ethylene-Co-Acrylic Lithium (Fluoro Sulfonyl)Imide), for All-Solid-State Li-Ion Batteries. ACS Appl. Mater. Interfaces 2019, 11 (38), 34930–34938. 10.1021/acsami.9b10474. 31469269

[ref61] Ahmed F.; Kim D.; Lei J.; Ryu T.; Yoon S.; Zhang W.; Lim H.; Jang G.; Jang H.; Kim W. UV-Cured Cross-Linked Astounding Conductive Polymer Electrolyte for Safe and High-Performance Li-Ion Batteries. ACS Appl. Mater. Interfaces 2021, 13 (29), 34102–34113. 10.1021/acsami.1c06233. 34261308

