# Supplementary material for: Argyrodite-Li6PS5Cl/Polymer-based Highly Conductive Composite Electrolyte for All-Solid-State Batteries
Source: ACS Appl Energy Mater. 2024 Feb 16;7(5):1842–53. doi: 10.1021/acsaem.3c02858 (PMC10934263; doi:10.1021/acsaem.3c02858)
Supplement: Supplementary file 1 — ae3c02858_si_001.pdf [file ae3c02858_si_001.pdf]

Supporting Information

Argyrodite–Li_6PS_5Cl/Polymer–based Highly Conductive Composite Electrolyte for All–Solid–State Batteries

Faiz Ahmed[a], Anna Chen[a], M. Virginia P. Altoé[b], Gao Liu[a]*

[a] Energy Storage and Distributed Resources Division, Lawrence Berkeley National Laboratory, Berkeley, California 94720, United States

[b] Molecular Foundry Division, Lawrence Berkeley National Laboratory, Berkeley, California 94720, United States

*Author to whom correspondence should be addressed: E-mail: gliu@lbl.gov (G. Liu).

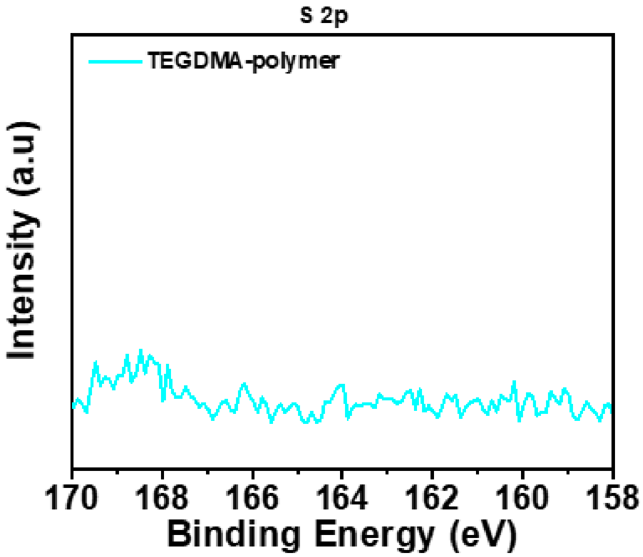

Figure S1: S 2p and P 2p XPS spectra of TEGDMA polymer.

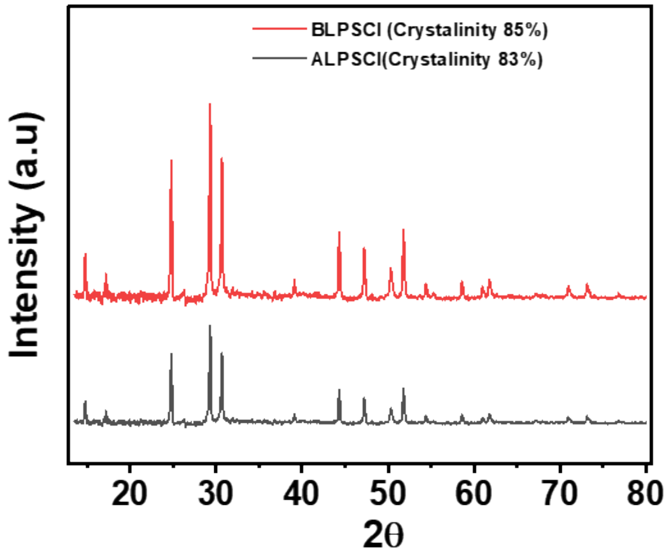

Figure S2: XRD spectra of ALPSCl and BLPSCl electrolytes.

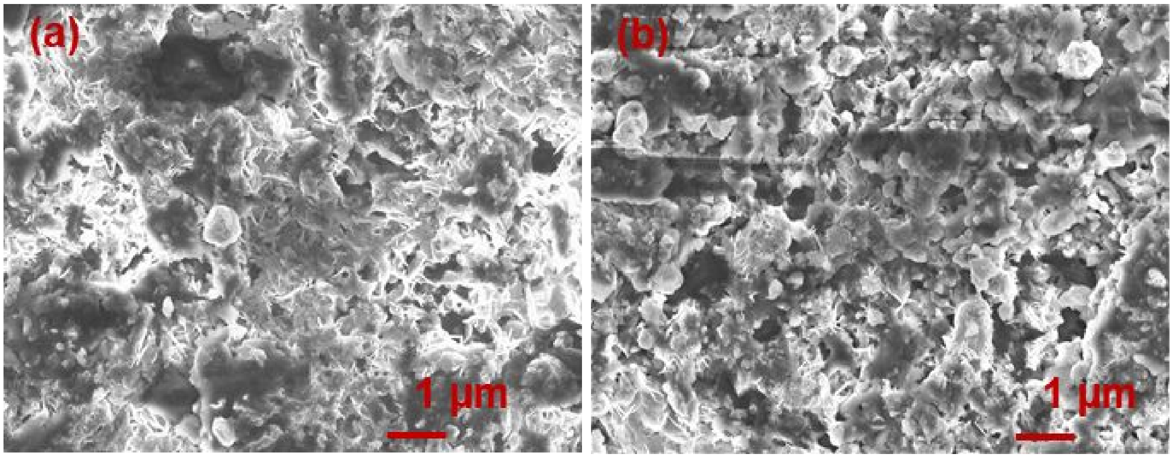

Figure S3: SEM images of (a) ALPSCl-P and (b) BLPSCl-P composite electrolytes.

Measurement of ionic conductivity

The σ of the SEs were measured based on the fitting results of the EIS plots according to the following equation (2).[1,2]

$$\sigma = \frac{l}{R_s a} \tag{1}$$

Where, l is the thickness of the SEs (i.e., the distance between the two symmetric SS electrodes), R_s is bulk resistance, and 'a' is the electrolyte area.

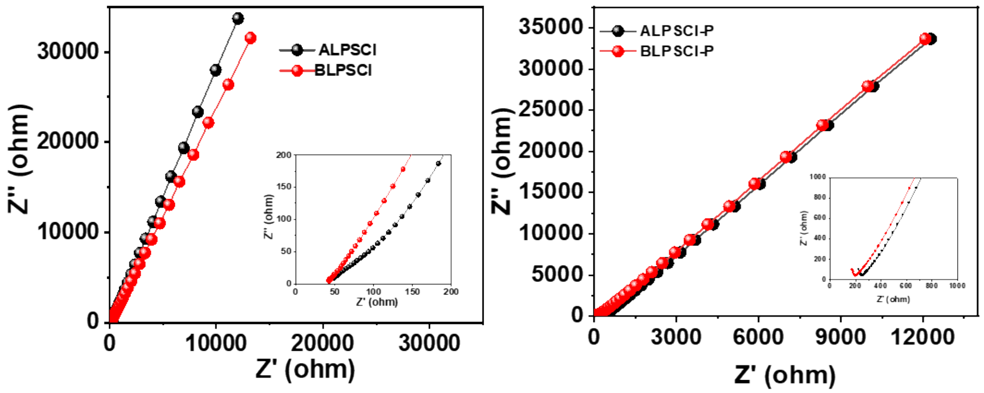

Figure S4: The fitted EIS spectra of the LPSCl and composite electrolytes at 25 ºC.

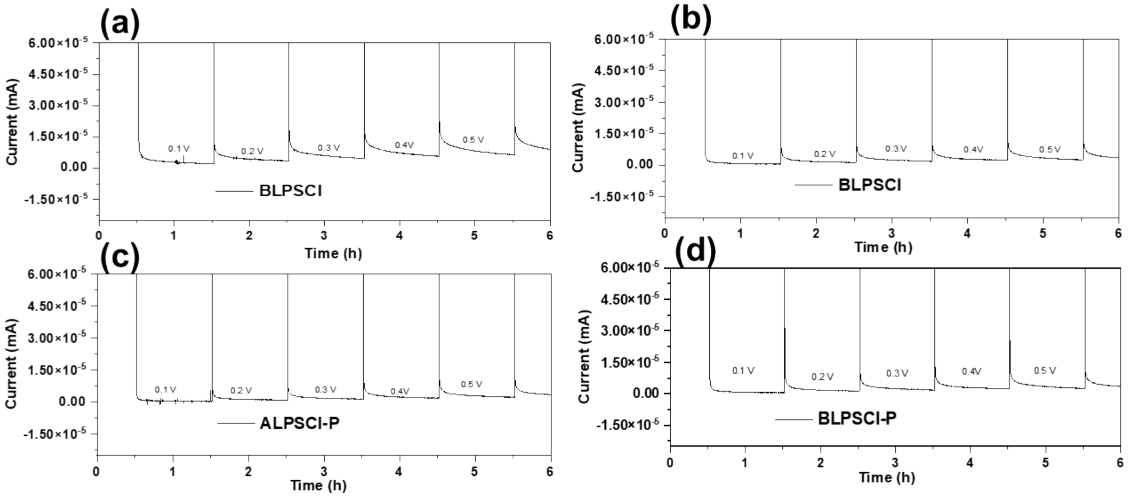

Figure S5: The current vs time curves at the range of (0.1–0.5) V of the SEs at 25 ºC.

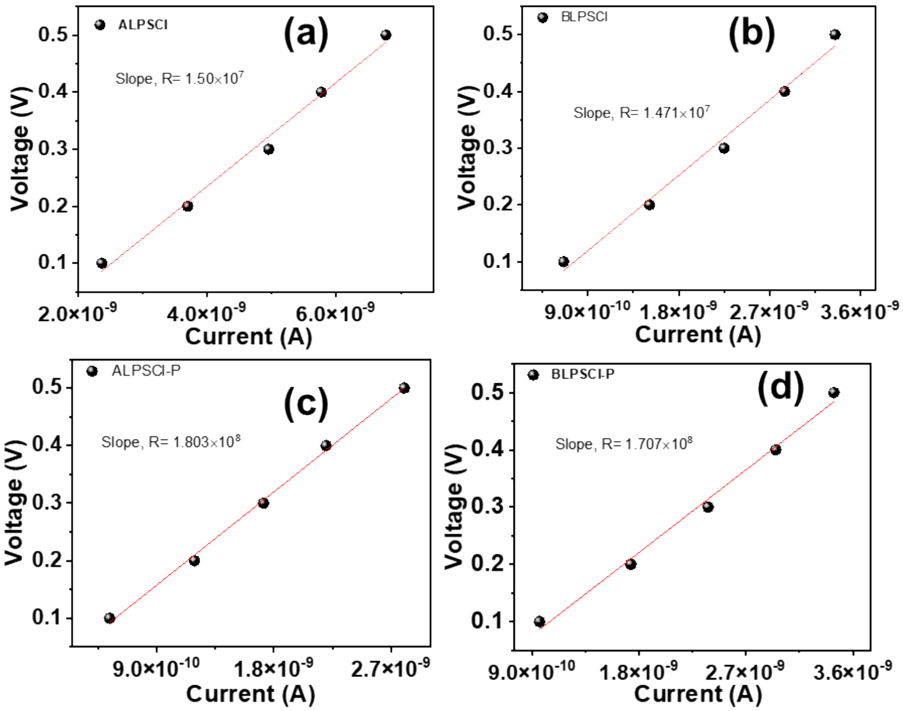

Figure S6: The voltage vs current curves of the SEs at 25 ºC.

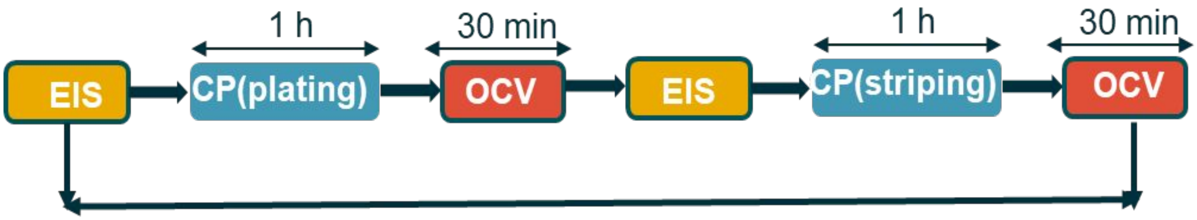

Figure S7: The experimental setup for the Li plating-striping of the SEs at 25 ºC

Table S1. The specific discharge capacity and Li-Li cycling stability of some recently reported composite SEs for ASSLBs.

Composite SE	Cathode	Discharge capacity (mAh/g)/ C rate	Li-Li Cycling stability/ Current density (mA/cm^2)	Ref.
PEGDMEL/LPSCl	Coated NMC811	85/0.1	500 h/0.1	[S3]
PVDF/LPSCl	-	-	65 h/0.2	[S4]
PEO/LPSCl	NMC811	47/0.05	1200 h/0.3	[S5]
PEO/LPSCl	$LiFePO_4$	80/0.2	180 h/0.4	[S6]
PEO/LPSCl	$LiFePO_4$	94/0.5	140 h/0.2	[S7]
PPO/LPSCl	-	-	150/0.1	[S8]
Li(G4)TFSI/LPSCl	NMC613	120/0.1	400 h/ 0.2	[S9]
**ALPSCl-P**	**Coated NMC811**	**134/0.1**	**1000 h/0.4**	**This work**
**BLPSCl-P**	**Coated NMC811**	**138/0.1**	**1000 h/0.4**	

Li(G4)TFSI = LiTFSI/tetraethylene glycol dimethyl ether polyacrylonitrile, LiTFSI= lithium bis(trifluoro-sulfonyl)imide, PEGDMEL= polyethylene glycol dimethyl ether, PVDF= poly(vinylidene difluride), PPO = Poly(p-phenylene oxide)

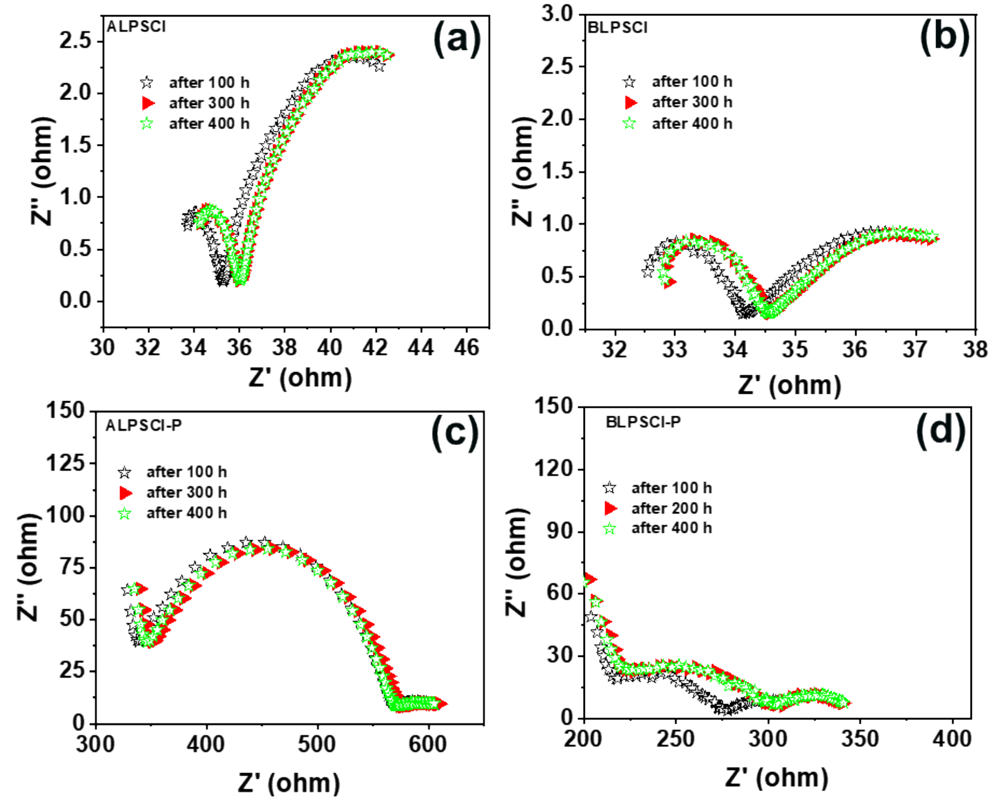

Figure S8: EIS spectra of (a) ALPSCl, (b) BLPSCl, (c) ALPSCl-P, and (b) BLPSCl-P electrolytes after different cycles.

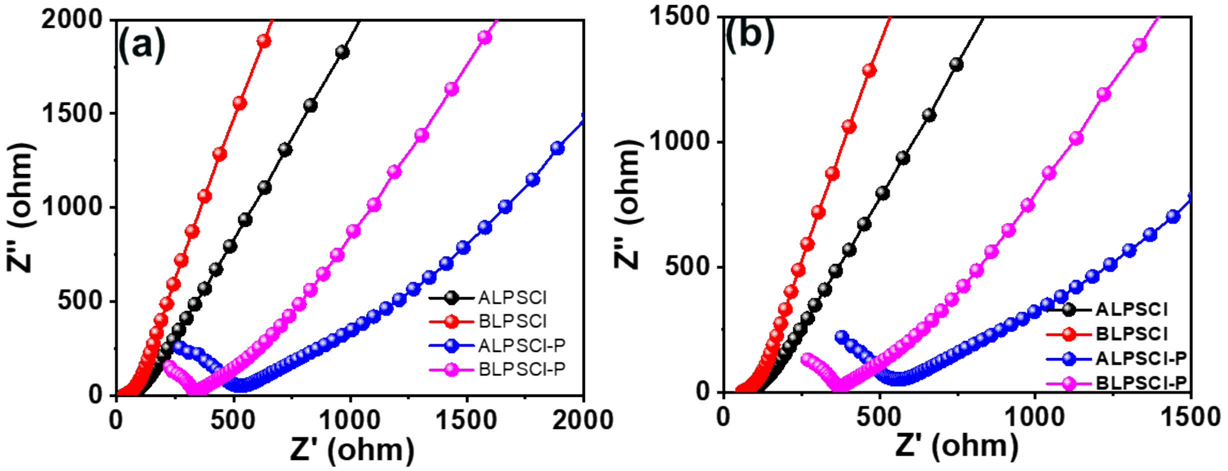

Figure S9: EIS spectra of SEs with the battery configuration of NMC811//SE//Li-In (a) before cycling (b) after 50 cycles.

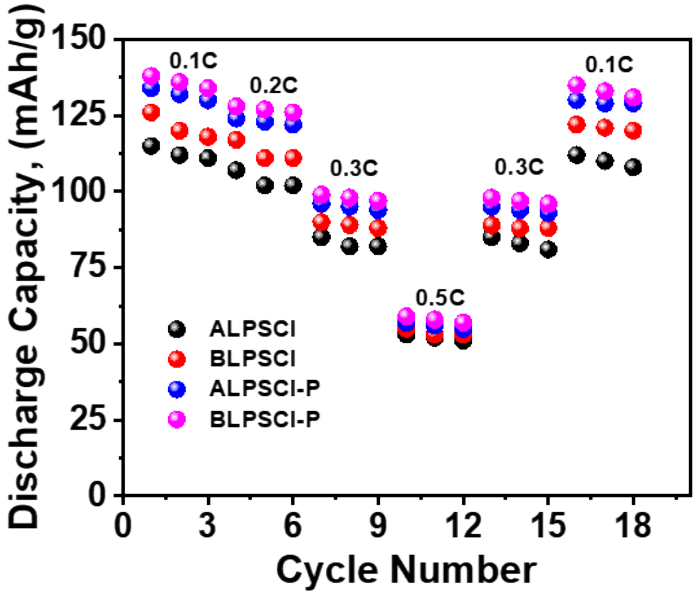

Figure S10: The rate capability of the cells with prepared SEs at various current densities from 0.1 C to 0.5 C at 25 ºC.

REFERENCES

(S1) Ahmed, F.; Choi, I.; Rahman, M. M.; Jang, H.; Ryu, T.; Yoon, S.; Jin, L.; Jin, Y.; Kim, W. Remarkable Conductivity of a Self–Healing Single–Ion Conducting Polymer Electrolyte, Poly(Ethylene–Co–Acrylic Lithium (Fluoro Sulfonyl)Imide), for All–Solid–State Li–Ion Batteries. *ACS Appl. Mater. Interfaces* **2019**, *11* (38), 34930–34938. https://doi.org/10.1021/acsami.9b10474.

(S2) Ahmed, F.; Kim, D.; Lei, J.; Ryu, T.; Yoon, S.; Zhang, W.; Lim, H.; Jang, G.; Jang, H.; Kim, W. UV–Cured Cross–Linked Astounding Conductive Polymer Electrolyte for Safe and High–Performance Li–Ion Batteries. *ACS Appl. Mater. Interfaces* **2021**, *13* (29), 34102–34113. https://doi.org/10.1021/acsami.1c06233.

(S3) Huo, H.; Jiang, M.; Mogwitz, B.; Sann, J.; Yusim, Y.; Zuo, T. T.; Moryson, Y.; Minnmann, P.; Richter, F. H.; Veer Singh, C.; Janek, J. Interface Design Enabling Stable Polymer/Thiophosphate Electrolyte Separators for Dendrite–Free Lithium Metal Batteries. *Angew. Chem. Inter. Ed.* **2023**, *62* (14). https://doi.org/10.1002/anie.202218044.

(S4) Wang, S.; Zhang, X.; Liu, S.; Xin, C.; Xue, C.; Richter, F.; Li, L.; Fan, L.; Lin, Y.; Shen, Y.; Janek, J.; Nan, C. W. High–Conductivity Free–Standing Li_6PS_5Cl/Poly(Vinylidene Difluoride) Composite Solid Electrolyte Membranes for Lithium–Ion Batteries. *J. Materiomics* **2020**, *6* (1), 70–76. https://doi.org/10.1016/j.jmat.2019.12.010.

(S5) Zhang, J.; Zheng, C.; Lou, J.; Xia, Y.; Liang, C.; Huang, H.; Gan, Y.; Tao, X.; Zhang, W. Poly(Ethylene Oxide) Reinforced Li_6PS_5Cl Composite Solid Electrolyte for All–Solid–State Lithium Battery: Enhanced Electrochemical Performance, Mechanical Property and Interfacial Stability. *J. Power Sources* **2019**, *412* (10), 78–85. https://doi.org/10.1016/j.jpowsour.2018.11.036.

(S6) Zou, C.; Yang, L.; Luo, K.; Liu, L.; Tao, X.; Yi, L.; Liu, X.; Luo, Z.; Wang, X. Preparation and Performances of Poly (Ethylene Oxide)–Li_6PS_5Cl Composite Polymer Electrolyte for All–Solid–State Lithium Batteries. *J. Electro. Chem.* **2021**, *900* (6), 115739. https://doi.org/10.1016/j.jelechem.2021.115739.

(S7) Zou, C.; Yang, L.; Luo, K.; Liu, L.; Tao, X.; Yi, L.; Liu, X.; Luo, Z.; Wang, X. Performance Improvement of Li_6PS_5Cl Solid Electrolyte Modified by Poly(Ethylene Oxide) –Based Composite Polymer Electrolyte with ZSM-5 Molecular Sieves. *ACS Appl. Energy Mater.* **2022**, *5* (2), 2356–2365. https://doi.org/10.1021/acsaem.1c03819.

(S8) Khomein, P.; Byeon, Y. W.; Liu, D.; Yu, J.; Minor, A. M.; Kim, H.; Liu, G. Lithium Phosphorus Sulfide Chloride–Polymer Composite via the Solution–Precipitation Process for Improving Stability toward Dendrite Formation of Li–Ion Solid Electrolyte. *ACS Appl. Mater. Interfaces* **2023**, *15* (9), 11723–11730. https://doi.org/10.1021/acsami.2c21302.

(S9) Zou, C.; Yang, L.; Luo, K.; Liu, L.; Tao, X.; Yi, L.; Liu, X.; Zhang, X.; Wang, X. In Situ Formed Protective Layer: Toward a More Stable Interface between the Lithium Metal Anode and Li_6PS_5Cl Solid Electrolyte. *ACS Appl. Energy Mater.* **2022**, *5* (7), 8428–8436. https://doi.org/10.1021/acsaem.2c00971.
